# Soldier Beetle Larvae Are Much More Common in the Fossil Record than Previously Anticipated

**DOI:** 10.3390/insects17040406

**Published:** 2026-04-09

**Authors:** Simon J. Linhart, Carolin Haug, Ana Zippel, Olympia Salvamoser, Patrick Müller, Joachim T. Haug

**Affiliations:** 1 Zoomorphology Group, Biocenter, Ludwig-Maximilians-Universität München (LMU Munich), Großhaderner Str. 2, Planegg-Martinsried, 82152 München, Germany; simon.linhart@palaeo-evo-devo.info (S.J.L.); carolin.haug@palaeo-evo-devo.info (C.H.);; 2 GeoBio-Center at LMU, Richard-Wagner-Str. 10, 80333 München, Germany; 3 School of Life Sciences, Technical University of Munich, Alte Akademie 8, 85354 Freising, Germany; 4 Independent Researcher, Kreuzbergstr. 90, 66482 Zweibrücken, Germany

**Keywords:** Cantharidae, Elateroidea, Coleoptera, Cretaceous, Eocene, Burmese amber, hypermetamorphosis

## Abstract

Soldier beetle larvae are characterised by numerous very small setae on their body surface, giving them a velvety appearance and hydrophobic properties, and paired gland openings on the segments of the thorax and abdomen for defensive purposes. Only recently, such a larva has been recognised in Cretaceous amber. Previously, only two specimens from Baltic amber were known from the fossil record. This provides the impression that such larvae are rare. We report 45 new larval specimens from Eocene Baltic amber and Cretaceous Kachin amber (Myanmar), demonstrating that these fossils are, in fact, more common, but simply not reported. We use the expanded dataset (with fossil and extant larvae and adults, more than 300 specimens) to explore the ontogeny of soldier beetles by quantitatively comparing morphological aspects over time. It appears that there is no major loss in the morphology of adults and larvae. On the contrary, certain larvae known in the modern fauna, so-called pre-larvae, seem absent in the fossil record. Due to the pre-larvae, some modern soldier beetles have certain characteristics of hypermetamorphosis. This type of developmental pattern is also characterised by a distinct change in the direction of the developmental trajectory. However, such a change could not be identified in soldier beetles.

## Simple Summary

Soldier beetle larvae can be recognised by a velvety surface and paired gland openings on the segments. Only three fossil specimens, all preserved in amber, have been reported so far, one from the Cretaceous and two from the Eocene. The small number would indicate that they are rare. We show 45 new larval specimens from the Cretaceous and the Eocene. This shows that soldier beetle larvae are not rare, but have not been reported. Together with specimens of the literature, we could create a dataset of over 300 specimens, fossil and extant, including adult and larval specimens. We investigated the development and the change over time in soldier beetles. To address this question, we compared certain body parts statistically. The results indicate no major loss in diversity over time. Only a specific larval stage, a so-called pre-larva, is not present in the fossil record so far. This pre-larva in some soldier beetles makes a difference compared to the development of most other beetles. The relation of this development to hypermetamorphosis, a development in which the first larvae are ecologically and morphologically different from the later larvae, and a non-direct development is present, is discussed. A hypermetamorphosis could not be identified in soldier beetles.

## 1. Introduction

The group of soldier beetles, Cantharidae, is well known for its colourful adults visiting flowers; therefore, it is more widely known to the public than many other beetle groups. The group is reasonably species-rich with more than 6700 formally described extant species [1] (table 1, p. 112) and is an ingroup of Elateroidea together with click beetles (Elateridae), false click beetles (Eucnemidae), fireflies (Lampyridae), or net-winged beetles (Lycidae), including the larvae of trilobite beetles, to name only some [2,3]. Like in many other larvae of Elateroidea, the larvae of soldier beetles are predatory, their mouthparts are facing forward (prognathous), and the lower jaws (maxilla) and the lower lip (labium) form a single compound structure (maxillo-labial complex [4,5] (p. 314)).

Soldier beetle larvae can be easily identified as such based on their dorsal surface, which has densely arranged tiny setae (“velvety appearance”; [5] (p. 314), [6] (p. 23)) providing hydrophobic properties, as well as a pair of prominent gland openings on each tergite. The openings are connected to defensive glands, which help the larva to deter predators [5,7,8]. Similar glands are also present in adults [8,9].

**Figure 1:**
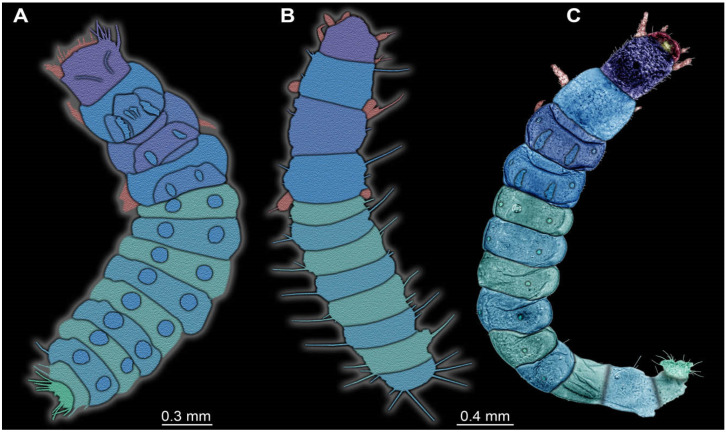
Soldier beetle larvae from the literature. (**A**) Colour-marked specimen, redrawn after [11] (p. 117, figure 252). (**B**) Colour-marked specimen, redrawn after [12] (p. 141, figure 2). (**C**) Colour-marked specimen from [13] (figure 2).

The fossil record of soldier beetles is largely restricted to adults, but these appear to be quite numerous (>230 formally described species [10]). Soldier beetle larvae appear to be a rarity in the fossil record, with only three putative records so far. One specimen was shown in a book on Baltic amber (about 40 million years old), but not determined beyond Coleoptera [11] (p. 117, figure 252). The specimen is partly verlumt (covered in whitish film), but the shape of the head and mandibles, as well as indications of the defensive gland openings, clearly point to this larva being a soldier beetle (Figure 1A). Another specimen from Baltic amber was depicted by Fowler [12] and identified as such by the original author (Figure 1B). A third specimen, this time from Kachin amber, Myanmar (about 100 million years old), has been recently reported, providing numerous details such as the tiny setae, the gland openings, and the details of the mouthparts (Figure 1C). This specimen demonstrated the presence of chemical defence in these beetle larvae 100 million years ago [13].

Within Elateroidea, there is a wide range of post-embryonic developmental patterns. Most soldier beetles have a “normal” type of holometabolous development, with a larval phase including several stages, followed by a pupa and adult. Yet, some ingroups of Cantharidae have early larvae that differ recognisably from the later ones, namely within Cantharinae and further within *Cantharis* and *Rhagonycha* ([14] (p. 102), [15]). Verhoeff [7,16] also indicates that the groups *Silis* and *Absidia (=Podistra)* would have pre-larvae, but no further support for the presence of pre-larvae in these groups could be found. Turis [17] mentioned that pre-larvae exist in *Podistra,* but draws his information from Verhoeff [7,16].

The morphologically differentiated larvae have been described as “pre-larva”, “prolarva”, “Передличинки” (translates roughly to “before larvae”) [17] or “Vorlarve”. They differ by not performing feeding, being filled with yolk, having rudimentary appendages, and a not-yet fully developed trachea system [14,15,17,18]. These characters also earned them the name “Fötusstadium” (≈“foetal stage” [14] (p. 102)).

Hypermetamorphosis describes a developmental pattern in which the usually ecologically uniform phase of the larva is separated into two distinct sub-phases, differing in behaviour and morphology [19,20]. Hypermetamorphosis has been coupled to different (qualitative) characteristics, especially the presence of an immobile pupa-like stage or a mobile triungulin (first) stage, and has been associated with a parasitic or parasitoid lifecycle [19,20,21,22]. Due to the differentiated larval phases in Cantharidae, their developmental pattern might be understood as a case of hypermetamorphosis. Fabre [23] already connected Cantharidae to hypermetamorphosis. However, he referred to the developmental pattern of “*Cantharis vesicatoria*” (Spanish fly). This species was originally described by Linnaeus [24] as “*Meloe vesicatoria*”, which has been identified as *Lytta vesicatoria*, a representative of Meloidae [25,26,27]. The confusion of the systematic position probably originated in the ambiguous use of the name “Cantharid(ae)” by Linnaeus and researchers of this time, by using “Cantharid” less strictly [e.g., p. 838, 21] and as a name for pharmacologically used beetles [28], even though the systematic difference was already recognised [25,28]. Verhoeff [14] also discussed the development of Cantharidae in *Cantharis rustica*, taking hypermetamorphosis into account. He concluded that Cantharidae are not undergoing hypermetamorphosis. However, the morphologically differing pre-larvae fulfil an important criterion for hypermetamorphosis.

Beetle larvae, in general and especially in the fossil record, are often underrepresented in the literature despite being, in fact, more common (see discussion in Linhart et al. [29]). This discrepancy is likely coupled to a focus on taxonomic aspects, which can be more easily dealt with on the adult side. For morpho-ecological considerations (though not only for these), larvae can be used and provide valuable information; for example, soldier beetle adults are rather short-lived [18,30], so most ecological interactions with the environment are performed by the larvae. As adult soldier beetles are quite numerous in amber, we expected that a detailed search should also lead to more finds of soldier beetle larvae in the same type of preservation. Here we report 45 additional soldier beetle larvae from Baltic and Kachin amber, and one adult specimen from Baltic amber. We also explore for the first time on a quantitative level if any developmental pattern within Cantharidae could be considered hypermetamorphic.

## 2. Materials and Methods

### 2.1. Materials

We report 46 new specimens preserved in amber interpreted as representatives of Cantharidae. In total, ten new larvae and one adult from Baltic amber, as well as 35 new larvae from Kachin amber, are reported. The adult specimen from Eocene Baltic amber is deposited in the collection of the Staatliches Museum für Naturkunde Karlsruhe, repository number SMNK-PAL 45612.

Four amber pieces with five larvae from Baltic amber are deposited in the collection of the Staatliche Naturwissenschaftliche Sammlungen Bayerns, Bayerische Staatssammlung für Paläontologie und Geologie, repository numbers SNSB-BSPG 2018 III 61, 67, 116 (with two individuals), and 245. Three pieces of Baltic amber are part of the Palaeo-Evo-Devo Research Group Collection of Arthropods, Ludwig-Maximilians-Universität München (LMU Munich), repository numbers PED 4026, 4028, and 4034. Two specimens from Baltic amber were not directly available for study, but images were kindly provided by traders (amberinclusions.eu, accessed on 1 April 2026, Jonas Damzen, Vilnius #13067; ambertreasure4u.com, accessed on 1 April 2026, Marius Veta, Vilnius #in-2680).

Five specimens from Cretaceous Kachin amber (Myanmar) are part of the collection of one of the authors (PM), repository numbers BUB 3017, 3738, 4780b, 5002, and 5163. Further 21 amber pieces from Kachin amber are part of the Palaeo-Evo-Devo Research Group Collection of Arthropods, Ludwig-Maximilians-Universität München (LMU Munich), repository numbers PED 0321, 0482 (with ten specimens), 1848, 2049, 2346, 2435, 2699, 3152, 3762, 3979, 4058, 4068, 4077, 4125, 4179, 4274, 4291, 4311, 4312, 4358, and 4394. Pieces in the PED collection were legally purchased on the trading platform eBay.com from various traders (amber-treasure-4u, burmite-miner, burmite-researcher, and goethgoeth) or on amberinclusions.eu (accessed on 1 April 2026, Jonas Damzen, Vilnius).

For quantitative comparison, images of specimens were used from the literature and the database BugGuide. Together with the newly reported specimens, this summed up to 127 extant larvae and pupae [5,6,7,8,14,15,17,18,31,32,33,34,35,36,37,38,39,40,41,42,43,44,45,46,47,48], 38 fossil larvae ([11,12,13], this contribution), 115 extant adults [5,18,46,48,49,50,51,52,53,54,55,56,57], and 23 fossil adults ([9,58,59,60,61,62,63,64,65,66,67,68,69,70,71], this contribution). In total, the dataset includes 303 specimens of Cantharidae for a quantitative morphological comparison (Supplementary Table S1).

### 2.2. Documentation and Image Processing

The new specimens were documented on a Keyence VHX-6000 digital microscope (Keyence, Osaka, Japan). Image enhancing (fusing stacks, merging image details, HDR) of the microscope was used, as well as different illuminations (ring or coaxial light) and backgrounds (white, black, glass) to get the best results. The amber pieces were prepared with glycerol and a cover slide on top to create an even surface (for further details, see [72,73]).

The images were further processed in Adobe Photoshop CS2 (Adobe, San José, CA, USA). This included optimising the contrast, sharpness, and histogram.

### 2.3. Outlines

Outlines of different structures were created in the vector graphics programme Inkscape (version 1.1; open source). Four different body parts were considered: the mandible (md), the head capsule (hc), the prothorax (pt), and the remaining thorax and abdomen (=trunk) (tr); these were combined in all adjacent combinations to ten types of shapes (see below). Other appendages, such as antennae, palps, or legs, were omitted. Mandibles were oriented for the outlines in the same way as in earlier studies [74,75,76,77]. For the adults, the closed elytra were included. Only the better-preserved half of the specimens was drawn and oriented as the right side (left side for mandible). Curved bodies were straightened step by step, by outlining each segment and rotating them into a straight orientation [78].

### 2.4. Analysis

Outlines were analysed by shape analysis (Elliptic Fourier analysis). The combination of the four body parts leads to ten datasets: hc+md (01); md (02); hc (03); full body (04); hc + pt + tr (05); md + hc + pt (06); hc + pt (07); pt (08); pt+tr (09); and tr (10). Dataset 01 included 241 specimens, 02 included 265 specimens, 03 included 266 specimens, 04 included 186 specimens, 05 included 205 specimens, 06 included 206 specimens, 07 included 225 specimens, 08 included 235 specimens, 09 included 213 specimens, 10 included 217 specimens. Datasets 01, 02, 04, 05, 06, 07, 09, and 10 were analysed using SHAPE (version 1.3; open source) [79]. Datasets 03 and 08 were analysed using R (Momocs [80]) due to difficulties with the alignment in SHAPE. The results of the shape analyses were plotted in OpenOffice Calc (version 4.1.15; open source) and redrawn in Adobe Photoshop CS2.

### 2.5. Measurements

Different measurements were performed using Inkscape. Lengths of the larvae were measured, where possible (all structures concerned needed to be preserved), from the anterior tip of the head capsule to the posterior end of the body along the median axis, taking the bending into account. In Inkscape, the bezier tool was used to draw a line along the distance of interest, and its length was measured. Then the real length of the line was calculated using the provided scale. The measurement can be influenced by the positioning of the line or the position of the specimen, resulting in difficulties in positioning the line. The length should at least give an indication of the size of the specimen, even though it might be inaccurate at the (second) decimal place.

## 3. Results

### 3.1. New Specimens of Soldier Beetles

The fossil record represented in the literature could be increased by 45 larval (Figure 2, Figure 3, Figure 4, Figure 5, Figure 6, Figure 7, Figure 8, Figure 9, Figure 10, Figure 11, Figure 12, Figure 13, Figure 14, Figure 15, Figure 16, Figure 17, Figure 18, Figure 19, Figure 20, Figure 21, Figure 22, Figure 23, Figure 24, Figure 25, Figure 26, Figure 27, Figure 28, Figure 29, Figure 30, Figure 31, Figure 32 and Figure 33) and one adult specimen (Figure 34). Of the larvae, 35 are preserved in Kachin (Myanmar) amber (Cretaceous). The other ten larval specimens are preserved in Baltic amber (Eocene). The single adult specimen is also preserved in Baltic amber. Some amber pieces include more than one specimen. PED 0482 includes ten specimens, and SNSB-BSPG 2018 III 116 includes two specimens. A detailed description of every specimen is provided as a descriptive matrix (cf. [81]; Supplementary Table S2).

### 3.2. General Observation on the Larvae

All newly described larval specimens have a morphology that identifies them as beetles. A maxillo-labial complex is present, and the mouthparts are forward-projecting, indicating that these are representatives of Elateroidea. Each mandible bears a tooth. All of the new specimens can be further identified as representatives of Cantharidae (soldier beetles) based on different characters. They possess paired openings on the segments of the thorax and abdomen (interpreted as those of defensive glands) and have a velvety surface (densely spaced small setae).

Furthermore, two types of character combinations can be recognised. The more abundant combination is that of a maxilla with three elements and the absence of a small forward projection on the median anterior part of the head capsule. The second combination consists of a maxilla with four elements and the presence of a small forward projection on the median anterior part of the head capsule. Both character combinations are present in Eocene (Baltic) and Cretaceous (Kachin) amber.

An additional differentiation of the specimens can be seen by body size. The smallest specimens are from a single amber piece with several specimens (PED 0482) and range from 0.66 to 1.18 mm in total length. Only one additional specimen is as small as the ones in PED 0482, namely a specimen from Baltic amber (SNSB-BSPG 2018 III 116, specimen 1). Additional specimens that are shorter than 2 mm are present in Baltic and Kachin amber (PED 3979, PED 4394, SNSB-BSPG 2018 III 61, and SNSB-BSPG 2018 III 67). Most of the specimens are in a size range between 2 and 5 mm. Only three specimens of Baltic and Kachin amber are longer than 5 mm (PED 2049, PED 2346, and PED 4028), and PED 2049 and PED 4028 are over 7 mm long.

### 3.3. Shape Analyses

All details of the shape analyses are available in Supplementary File S1.

Dataset 01, head capsule and mandible (Figure 35A): The analysis resulted in eight effective PCs summarising 91.64% of the overall variation. PC1 summarises 33.20% of the overall variation. It is mainly influenced by the anterior median part of the head capsule (labrum) and the broadness of the mandible. A negative value represents an extended anterior part of the head capsule and a narrower mandible. A positive value represents the absence of the extended anterior part of the head capsule and a broader mandible.

PC2 summarises 27.87% of the overall variation. It is mainly influenced by the anterior structures of the head capsule. A negative value represents a distinctly separated mandible from the head capsule. A positive value represents a mandible partly hidden by the anterior structures of the head capsule.

Dataset 02, mandible (Figure 35B): The analysis resulted in six effective PCs summarising 93.88% of the overall variation. PC1 summarises 46.61% of the overall variation. It is mainly influenced by the bending of the mandible. A negative value represents a relatively straight mandible. A positive value represents a bent mandible.

PC2 summarises 24.86% of the overall variation. It is mainly influenced by the presence or absence of a tooth; a negative value represents a mandible without a tooth, and a positive value represents a mandible with a tooth.

Dataset 03, head capsule (Figure 36A): The analysis resulted in six effective PCs summarising 92.27% of the overall variation. PC1 summarises 37.39% of the overall variation. It is mainly influenced by the broadness of the head capsule. A negative value represents a broad head capsule. A positive value represents a narrow head capsule.

PC2 summarises 26.29% of the overall variation. It is mainly influenced by the lateral side of the head capsule. A negative value represents an outwardly curved lateral side of the head capsule. A positive value represents an inwardly curved lateral side of the head capsule.

Dataset 04, full body (Figure 36B): The analysis resulted in seven effective PCs summarising 89.64% of the overall variation. PC1 summarises 54.09% of the overall variation. It is mainly influenced by the anterior part of the head capsule and the prothorax. A negative value represents an anterior extension on the head capsule (labrum) and a distinctly recognisable prothorax. A positive value represents the absence of an anterior extension on the head capsule (labrum) and a more smoothly embedded prothorax.

PC2 summarises 13.30% of the overall variation. It is mainly influenced by the anterior part of the body. A negative value represents a broad prothorax and head capsule, and a slightly forward-pointing anterior part of the head capsule. A positive value represents a smooth transition from the prothorax toward the head capsule and a slightly inward curved anterior part of the head capsule.

Dataset 05, full body without mandible (Figure 37A): The analysis resulted in seven effective PCs summarising 91.00% of the overall variation. PC1 summarises 55.92% of the overall variation. It is mainly influenced by the head capsule and the prothorax. A negative value represents a head capsule with an anterior forward projecting structure (labrum), a distinct lateral extension (eyehill, eyesocket), and a distinctly recognisable prothorax. A positive value represents a rounded, slightly elongated head capsule and a smooth transition over the region of the prothorax.

PC2 summarises 14.55% of the overall variation. It is mainly influenced by the anterior part of the body. A negative value represents a distinctly separable head capsule and prothorax, and a triangular anterior part of the head capsule. A positive value represents a smooth transition of the head capsule and prothorax, and a rounded anterior part of the head capsule.

Dataset 06, head capsule, mandible, and prothorax (Figure 37B): The analysis resulted in nine effective PCs summarising 89.98% of the overall variation. PC1 summarises 26.07% of the overall variation. It is mainly influenced by the position of the mandible. A negative value represents a distinctly separable mandible positioned on the outer (more lateral) part of the head capsule. A positive value represents a mandible that is partially concealed by other anterior structures and positioned more medially on the head capsule.

PC2 summarises 21.49% of the overall variation. It is mainly influenced by the anterior and slightly by the posterior region. A negative value represents a large mandible with a more square-shaped head capsule and a posterior, more straight ending of the prothorax. A positive value represents a more triangular head capsule (with distinctly recognisable labrum and eyehill), a smaller, more median positioned mandible, and a rounded posterior end of the prothorax.

Dataset 07, head capsule and prothorax (Figure 38A): The analysis resulted in eight effective PCs summarising 90.53% of the overall variation. PC1 summarises 28.36% of the overall variation. It is mainly influenced by the shape of the head capsule and the posterior end of the prothorax. A negative value represents a triangular head capsule (with distinctly recognisable labrum and eyehill) and a rounded posterior end of the prothorax. A positive value represents a more square-shaped head capsule and a straight posterior end of the prothorax.

PC2 summarises 19.25% of the overall variation. It is mainly influenced by the transition between the head capsule and the prothorax, as well as the anterior rim of the head capsule. A negative value represents a more distinct separation between the head capsule and prothorax, as well as a rounded anterior rim of the head capsule. A positive value represents a smoother transition between the head capsule and prothorax, and a more forward-pointing anterior rim of the head capsule.

Dataset 08, prothorax (Figure 38B): The analysis resulted in six effective PCs summarising 95.00% of the overall variation. PC1 summarises 56.54% of the overall variation. It is mainly influenced by the broadness of the prothorax. A negative value represents a narrow prothorax. A positive value represents a broad prothorax.

PC2 summarises 17.41% of the overall variation. It is mainly influenced by the shape of the lateral side of the prothorax. A negative value represents a rounded and a positive value a square-shaped prothorax.

Dataset 09, thorax and abdomen (=trunk) (Figure 39A): The analysis resulted in six effective PCs summarising 91.01% of the overall variation. PC1 summarises 60.40% of the overall variation. It is mainly influenced by the anterior part of the prothorax and the lateral side of the remaining thorax and abdomen (=trunk). A negative value represents an anteriorly rounded prothorax and a straight lateral side (≈elytra). A positive value represents a broader, anteriorly straight prothorax and a more uneven lateral side.

PC2 summarises 12.72% of the overall variation. It is mainly influenced by the transition between the prothorax and the remaining thorax and abdomen (=trunk). A negative value represents a distinct separation between the prothorax and the remaining thorax and abdomen (=trunk). A positive value represents a smooth transition between the prothorax and the remaining thorax and abdomen (=trunk).

Dataset 10, thorax and abdomen (=trunk) without prothorax (Figure 39B): The analysis resulted in six effective PCs summarising 92.58% of the overall variation. PC1 summarises 66.47% of the overall variation. It is mainly influenced by the shape of the lateral side and by the anterior part of the trunk without the prothorax. A negative value represents an even lateral side and a concave anterior end (≈elytra). A positive value represents an uneven lateral side and a straight anterior end of the trunk without the prothorax.

PC2 summarises 10.59% of the overall variation. It is mainly influenced by the posterior end of the body. A negative value represents a broader blunt end. A positive value represents a more pointed posterior end of the body.

### 3.4. Developmental Stages

The PC1 of datasets 01 (head capsule and mandible), 02 (mandible), 03 (head capsule), and 10 (thorax and abdomen (=trunk) without prothorax) were plotted against the developmental stages (extant: early larva or pre-larva, late larva, pupa, adult; fossil: larva and adult; Figure 40). For the head capsule and mandible (Figure 40A), the specimens show a diversification towards the adult stage along PC1. Fossils are within the distribution of the extant counterparts. For the mandible (Figure 40B), the specimens show a shift from the pre-larvae towards the adult stage, most extreme from pre-larvae to later larvae. Fossils are roughly within the distribution of the extant counterparts. For the head capsule (Figure 40C), the specimens show a small shift from pre-larvae to later larvae and a diversification towards the adult stage. Fossils are within the distribution of the extant counterparts. For the thorax and abdomen (=trunk) without the prothorax (Figure 40D), the specimens show a diversification from pre-larvae to later larvae and a shift towards the adults. Fossils are roughly within the distribution of the extant counterparts; only one adult specimen clearly increases the occupied morphospace of the adult fossils.

## 4. Discussion

### 4.1. Identity of New Larval Specimens: Cantharidae

**Figure 2:**
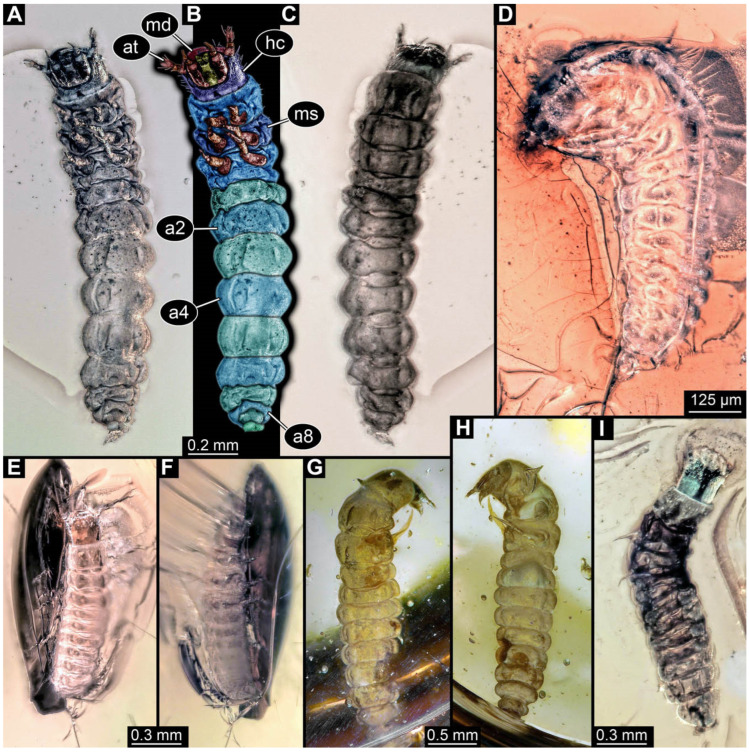
New soldier beetle larvae from Baltic amber; all amber pieces bear the repository number SNSB-BSPG 2018 III, followed by a specific number for each amber specimen. (**A**–**C**) Specimen 67: (**A**) ventral view; (**B**) colour-marked version of (**A**); (**C**) dorsal view. (**D**) Specimen 116, individual 2, oblique ventral view. (**E**,**F**) Specimen 116, individual 1: (**E**) dorsal view; (**F**) ventral view. (**G**,**H**) Specimen 245: (**G**) dorsal view; (**H**) ventral view. (**I**) Specimen 61, dorsal view. Abbreviations: a2–8 = abdomen segments 2–8; at = antenna; hc = head capsule; md = mandible; ms = mesothorax.

**Figure 3:**
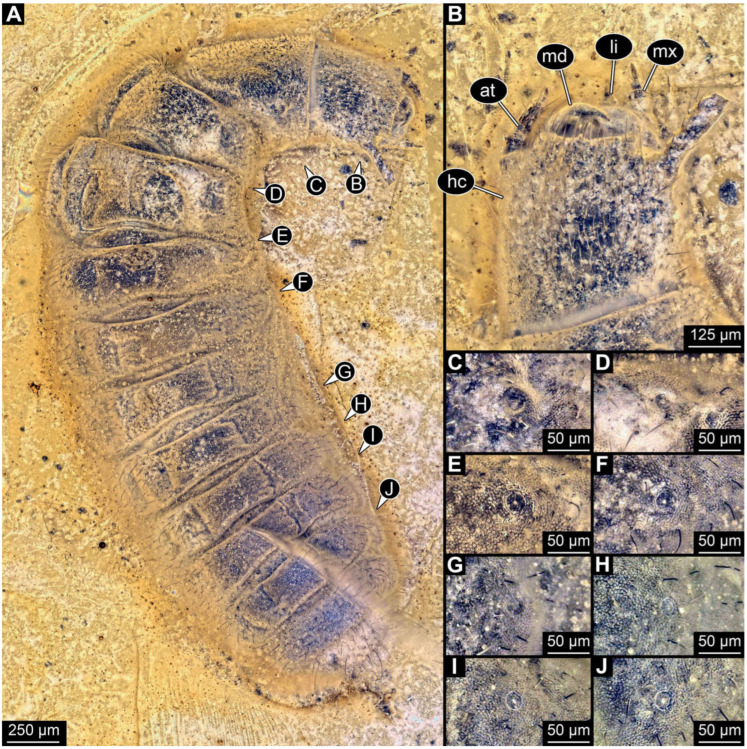
New soldier beetle larva from Baltic amber, PED 4026. (**A**) Dorsal view. (**B**) Head detail, dorsal view. (**C**–**J**) Defensive glands details. Abbreviations: at = antenna; hc = head capsule; li = labium; md = mandible; mx = maxilla.

**Figure 4:**
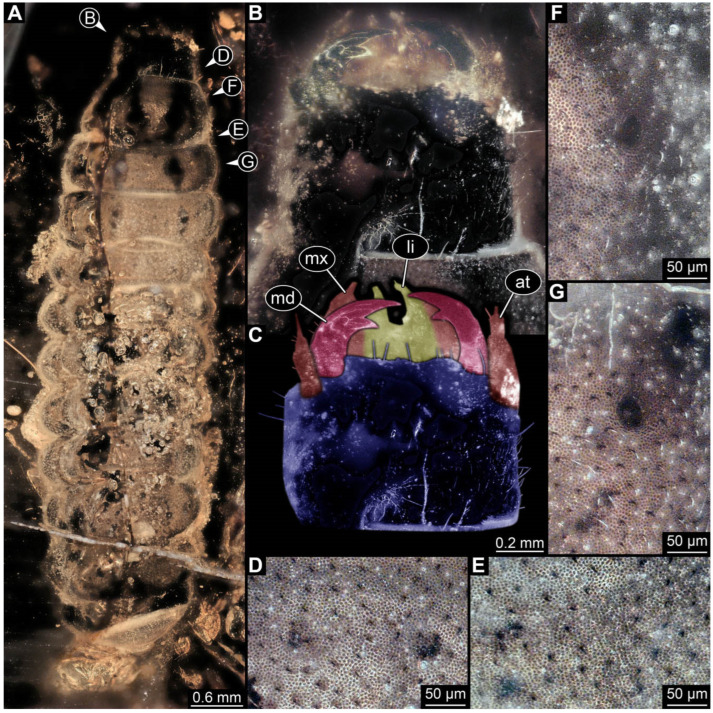
New soldier beetle larva from Baltic amber, PED 4028. (**A**) Dorsal view. (**B**) Head detail, dorsal view. (**C**) Colour-marked version of (**B**). (**D**,**E**) Velvety surface details. (**F**,**G**) Defensive glands details. Abbreviations: at = antenna; li = labium; md = mandible; mx = maxilla.

**Figure 5:**
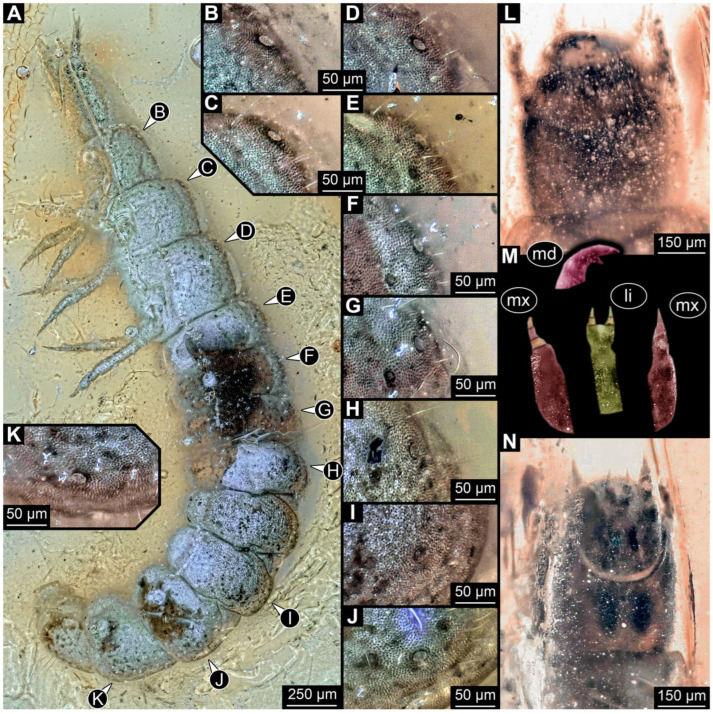
New soldier beetle larva from Baltic amber, PED 4034. (**A**) Lateral view. (**B**–**K**) Defensive glands details. (**L**) Head detail, dorsal view. (**M**) Mouthparts colour-marked. (**N**) Head detail, ventral view. Abbreviations: li = labium; md = mandible; mx = maxilla.

**Figure 6:**
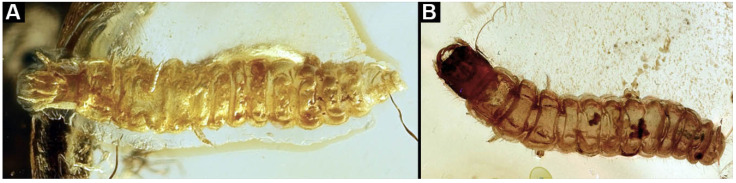
New soldier beetle larvae from Baltic amber. (**A**) Ventral view, image kindly provided by Jonas Damzen, Vilnius (amberinclusions.eu, #13067). (**B**) Dorsal view, image kindly provided by Marius Veta, Vilnius (ambertreasure4u.com, #in-2680).

**Figure 7:**
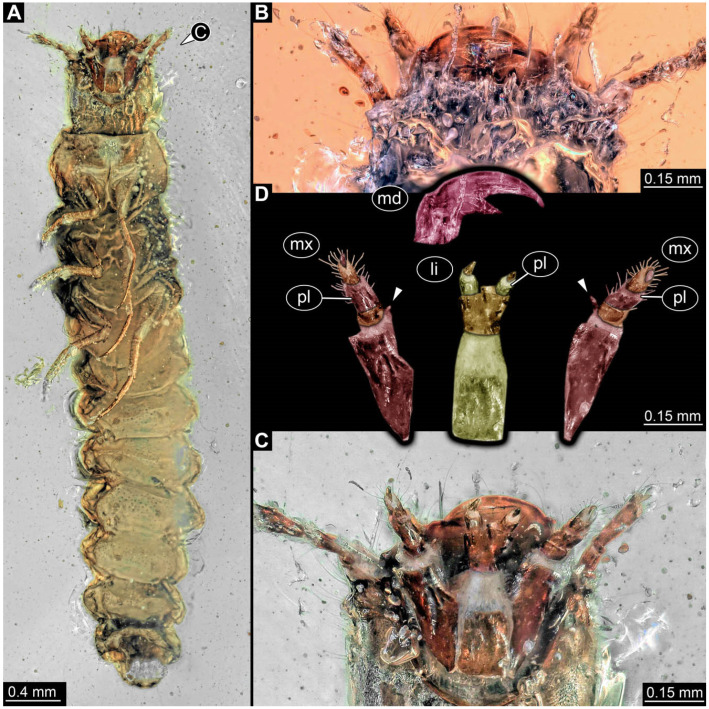
New soldier beetle larva from Kachin amber, Myanmar, PED 0321. (**A**) Ventral view. (**B**) Mouthparts detail, dorsal view. (**C**) Mouthparts detail, ventral view. (**D**) Mouthparts colour-marked, arrows mark endites (galea). Abbreviations: li = labium; md = mandible; mx = maxilla; pl = palp.

**Figure 8:**
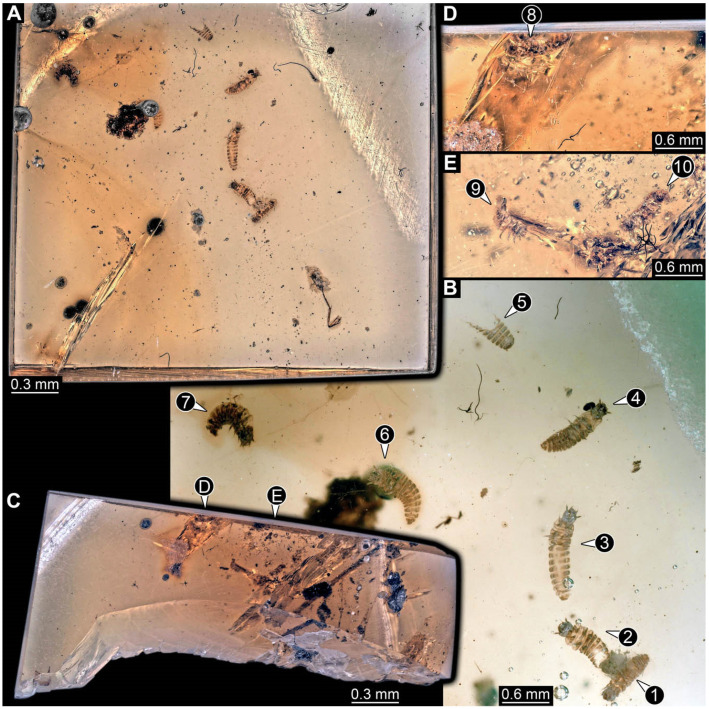
New soldier beetle larvae from Kachin amber, Myanmar, PED 0482, amber broken in two pieces. (**A**) Overview of a larger amber piece with several larvae. (**B**) Close-up of (**A**) with larvae numbered. (**C**) Overview of a smaller amber piece. (**D**) Close-up on specimen 8. (**E**) Close-up on specimens 9 and 10.

**Figure 9:**
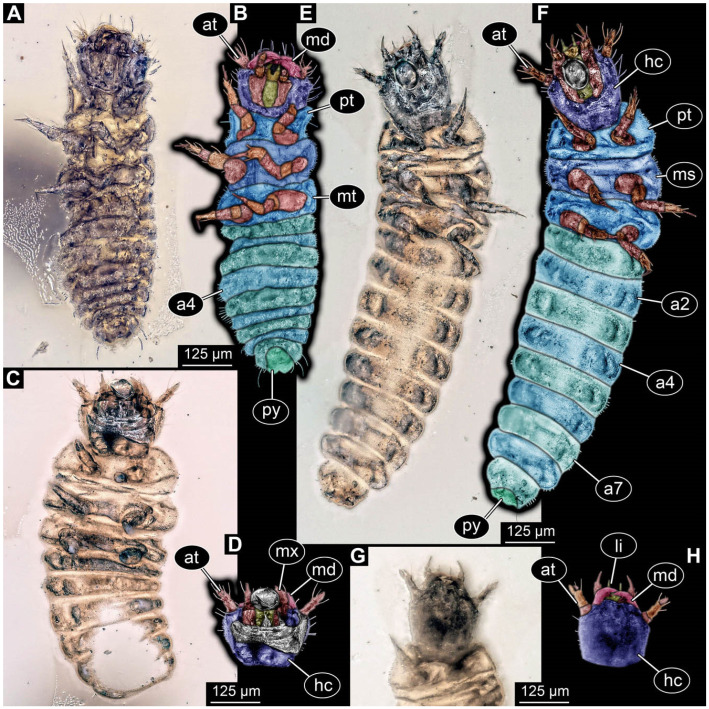
New soldier beetle larvae from Kachin amber, Myanmar, PED 0482, continued. (**A**,**B**) Specimen 1, ventral view. (**C**,**D**) Specimen 2, ventral view. (**E**–**H**) Specimen 3. (**E**,**F**) Ventral view. (**G**,**H**) Dorsal view of head capsule. Abbreviations: a2–7 = abdomen segments 2–7; at = antenna; hc = head capsule; li = labium; md = mandible; ms = mesothorax; mt = metathorax; mx = maxilla; pt = prothorax; py = pygopod.

**Figure 10:**
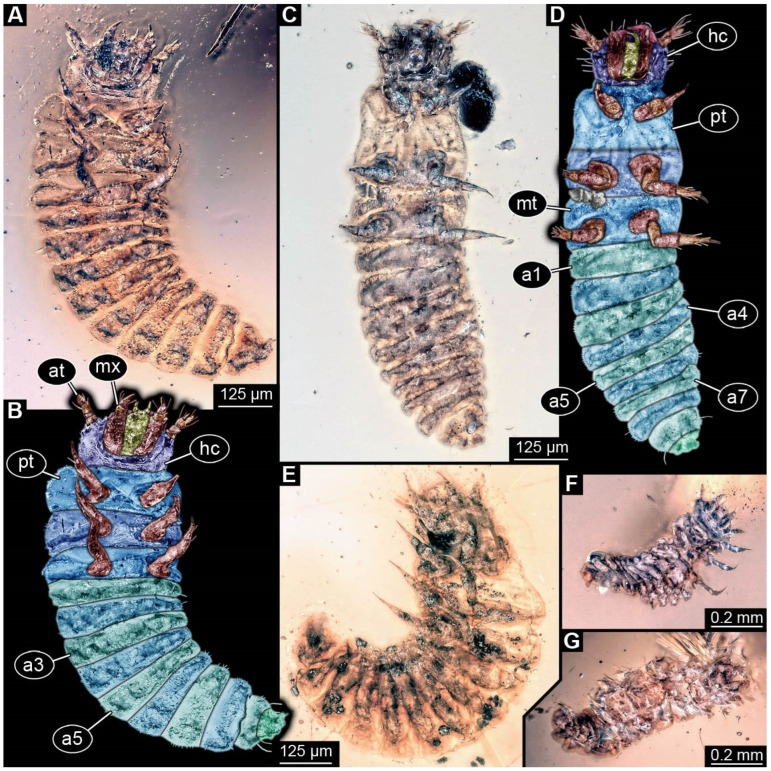
New soldier beetle larvae from Kachin amber, Myanmar, PED 0482, continued. (**A**,**B**) Specimen 6, ventral view. (**C**,**D**) Specimen 4, ventral view. (**E**) Specimen 7, latero-ventral view. (**F**) Specimen 9, ventral view. (**G**) Specimen 10, ventral view. Abbreviations: a1–7 = abdomen segments 1–7; at = antenna; hc = head capsule; mt = metathorax; mx = maxilla; pt = prothorax.

**Figure 11:**
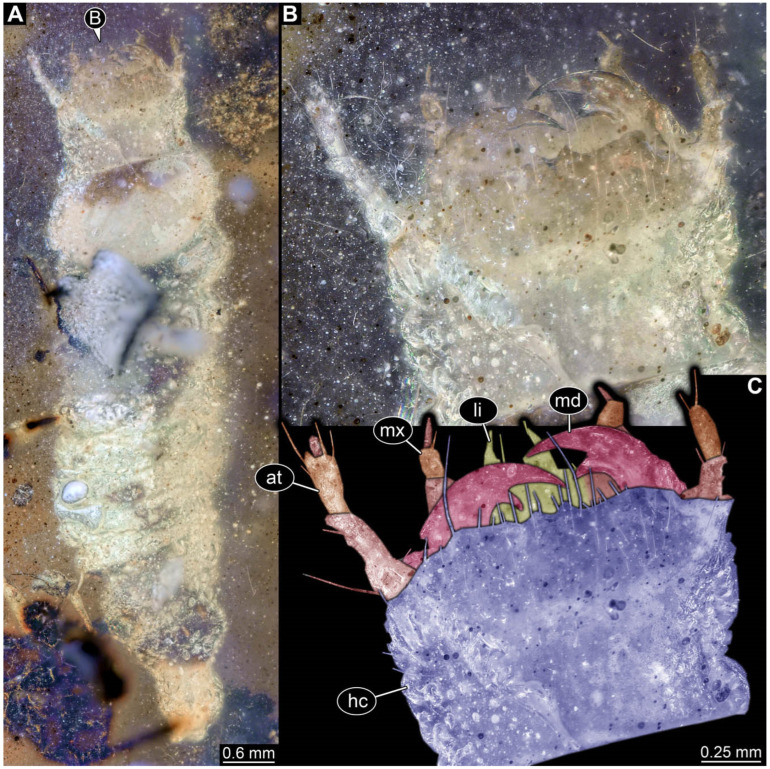
New soldier beetle larva from Kachin amber, Myanmar, PED 2049. (**A**) Dorsal view. (**B**) Head detail, dorsal view. (**C**) Colour-marked version of (**B**). Abbreviations: at = antenna; hc = head capsule; li = labium; md = mandible; mx = maxilla.

**Figure 12:**
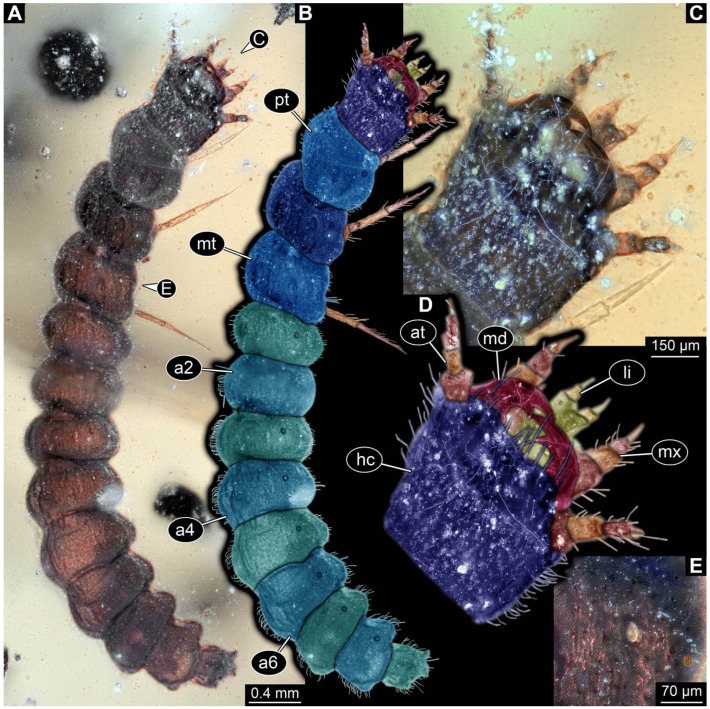
New soldier beetle larva from Kachin amber, Myanmar, PED 2346. (**A**) Dorsal view. (**B**) Colour-marked version of (**A**). (**C**) Head detail, dorsal view. (**D**) Colour-marked version of (**C**). (**E**) Defensive gland detail. Abbreviations: a2–6 = abdomen segment 2–6; at = antenna; hc = head capsule; li = labium; md = mandible; mt = metathorax; mx = maxilla; pt = prothorax.

**Figure 13:**
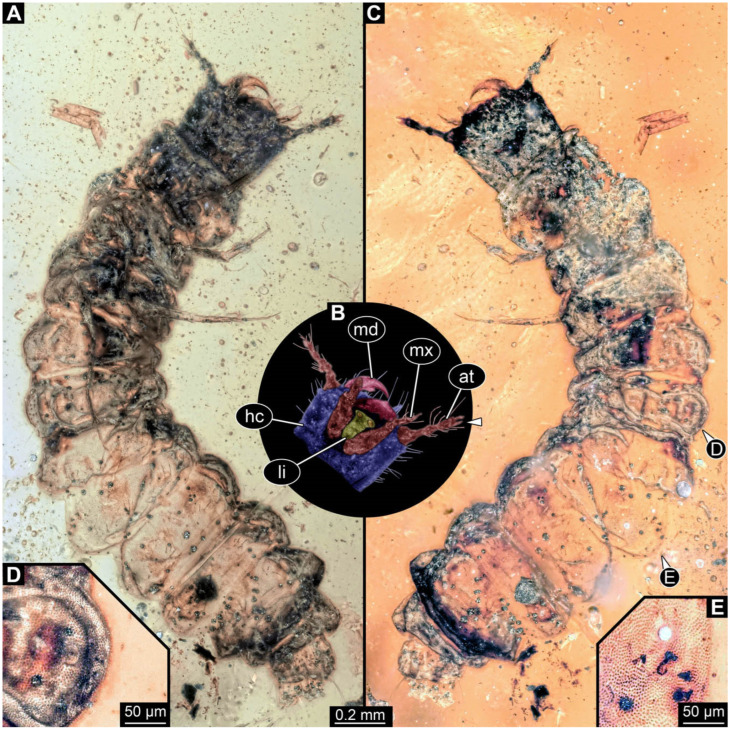
New soldier beetle larva from Kachin amber, Myanmar, PED 2699. (**A**) Ventral view. (**B**) Colour-marked version of the head. (**C**) Dorsal view. (**D**) Defensive gland detail. (**E**) Velvety surface detail. Abbreviations: at = antenna; hc = head capsule; li = labium; md = mandible; mx = maxilla.

**Figure 14:**
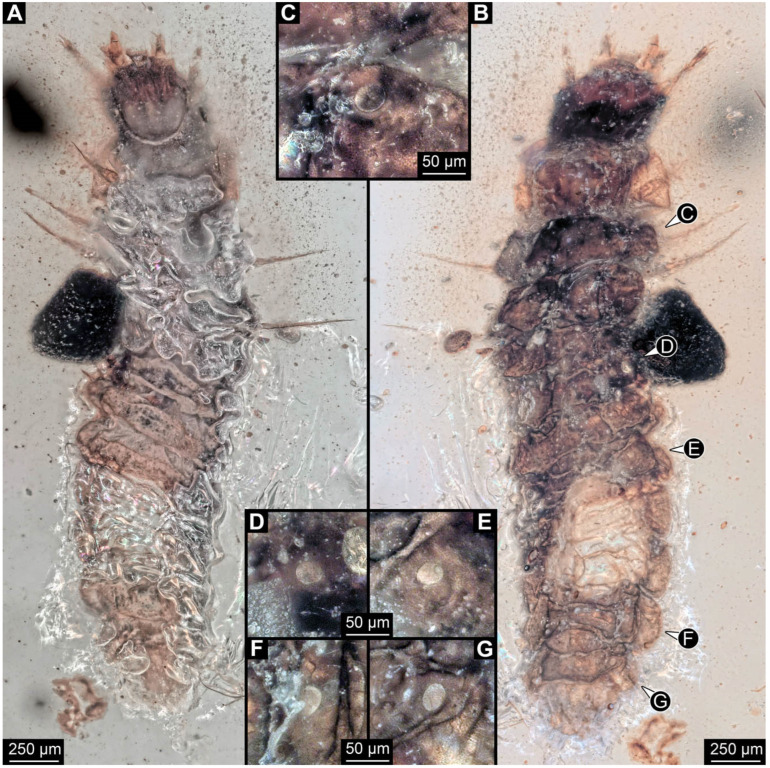
New soldier beetle larva from Kachin amber, Myanmar, PED 3152. (**A**) Ventral view. (**B**) Dorsal view. (**C**–**G**) Defensive glands details.

**Figure 15:**
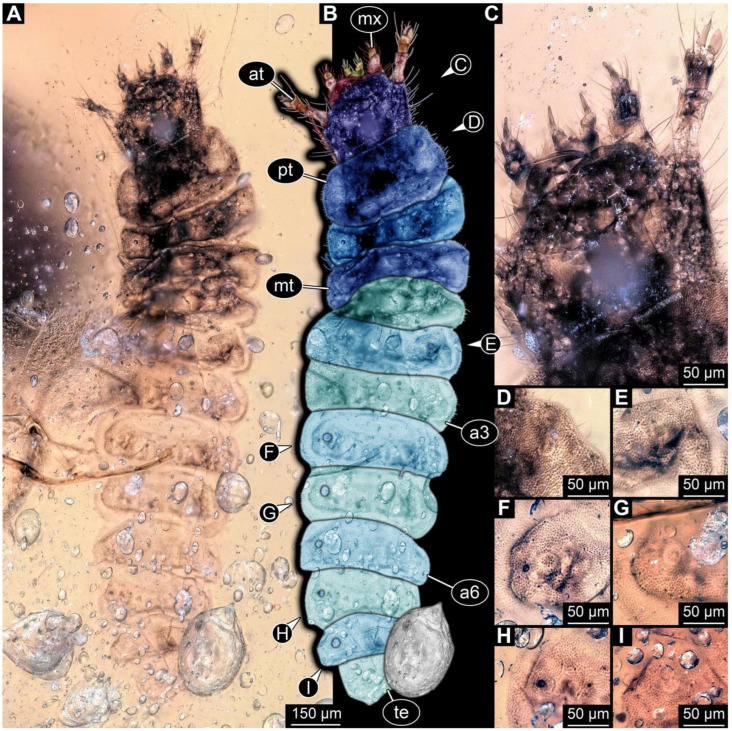
New soldier beetle larva from Kachin amber, Myanmar, PED 3979. (**A**) Dorsal view. (**B**) Colour-marked version of (**A**). (**C**) Head detail, dorsal view. (**D**–**I**) Defensive glands details. Abbreviations: a3–6 = abdomen segments 3–6; at = antenna; mt = metathorax; mx = maxilla; pt = prothorax; te =trunk end (posterior end of abdomen).

**Figure 16:**
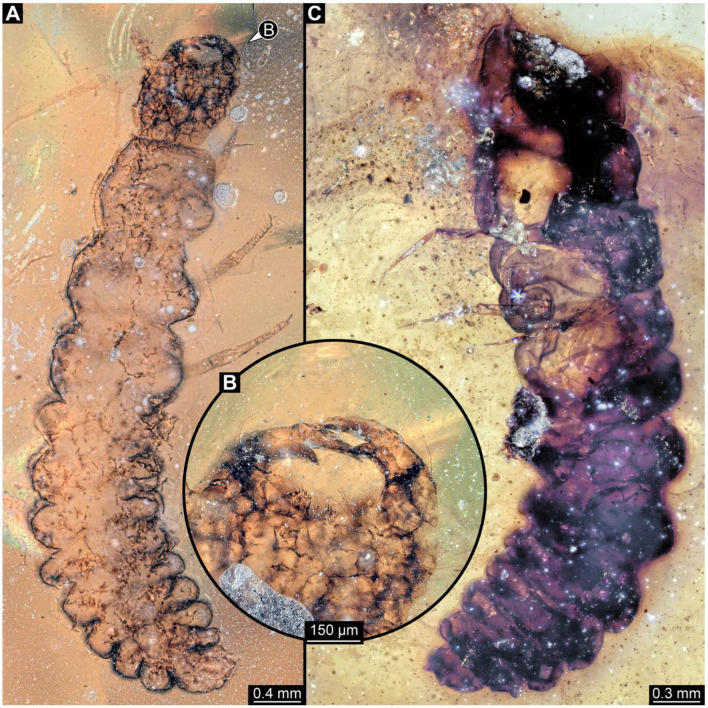
New soldier beetle larvae from Kachin amber, Myanmar. (**A,B**) PED 3762. (**A**) Dorsal view. (**B**) Mandible detail. (**C**) PED 1848, dorsal view.

**Figure 17:**
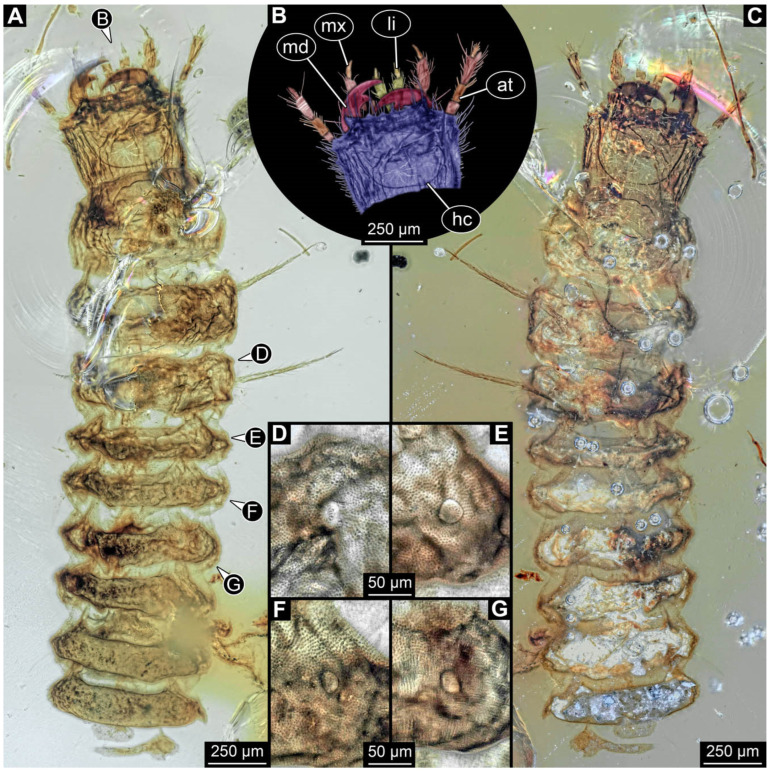
New soldier beetle larva from Kachin amber, Myanmar, PED 4058. (**A**) Dorsal view. (**B**) Colour-marked version of the head. (**C**) Ventral view. (**D**–**G**) Defensive glands details. Abbreviations: at = antenna; hc = head capsule; li = labium; md = mandible; mx = maxilla.

**Figure 18:**
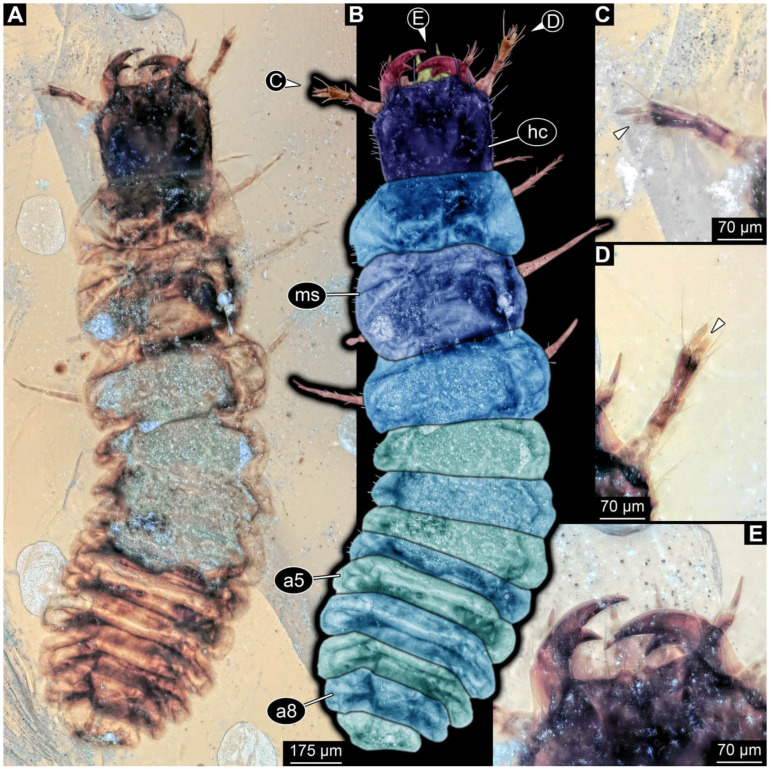
New soldier beetle larva from Kachin amber, Myanmar, PED 4068. (**A**) Dorsal view. (**B**) Colour-marked version of (**A**). (**C**,**D**) Antenna details, arrows mark sensory structures. (**E**) Mandible detail. Abbreviations: a5–8 = abdomen segment 5–8; hc = head capsule; ms = mesothorax.

**Figure 19:**
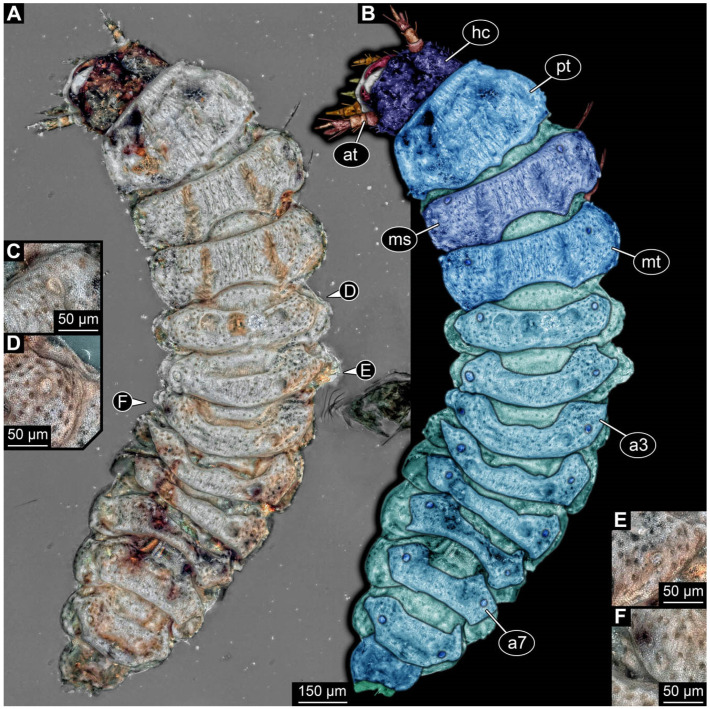
New soldier beetle larva from Kachin amber, Myanmar, PED 4077. (**A**) Dorsal view. (**B**) Colour-marked version of (**A**). (**C**–**F**) Defensive glands details. Abbreviations: a3–7 = abdomen segment 3–7; at = antenna; hc = head capsule; ms = mesothorax; mt = metathorax; pt = prothorax.

**Figure 20:**
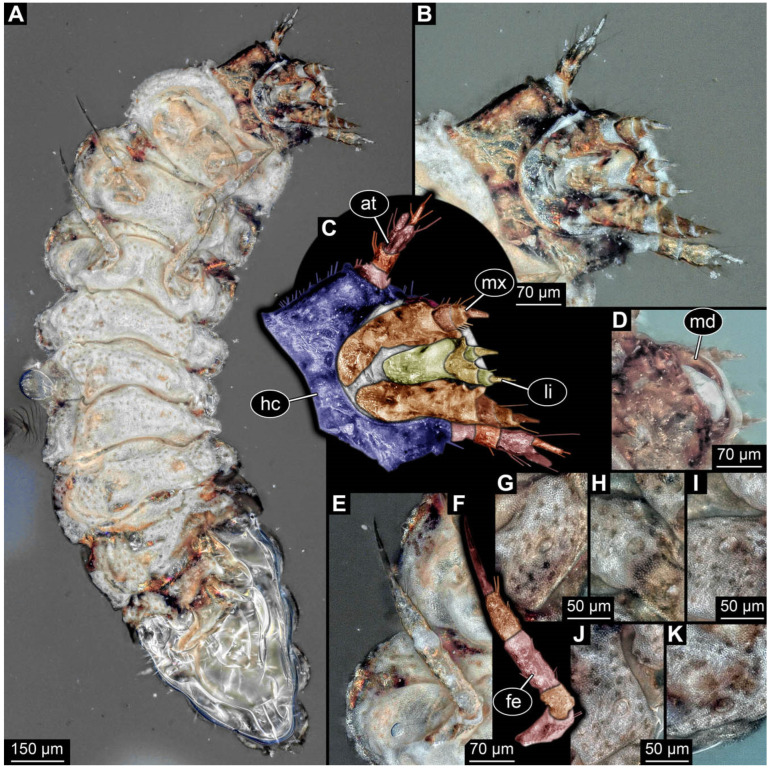
New soldier beetle larva from Kachin amber, Myanmar, PED 4077, continued. (**A**) Ventral view. (**B**) Head detail, ventral view. (**C**) Colour-marked version of (**B**). (**D**) Head detail, dorsal view. (**E**) Leg detail. (**F**) Colour-marked version of (**E**). (**G**–**K**) Defensive glands details. Abbreviations: at = antenna; fe = femur; hc = head capsule; li = labium; md = mandible; mx = maxilla.

**Figure 21:**
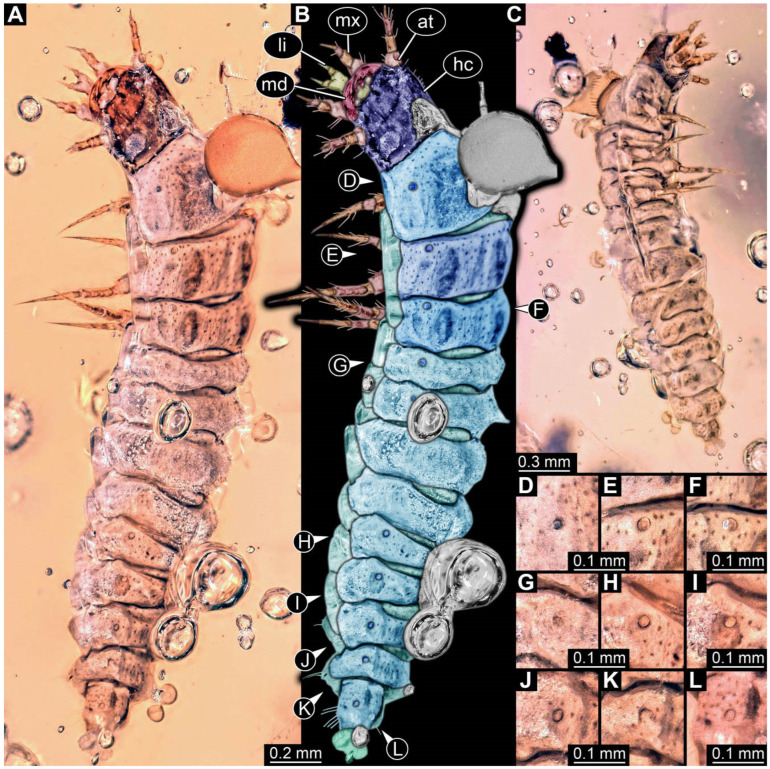
New soldier beetle larva from Kachin amber, Myanmar, BUB 3017. (**A**) Dorsal view. (**B**) Colour-marked version of (**A**). (**C**) Ventral view. (**D**–**L**) Defensive glands details. Abbreviations: at = antenna; hc = head capsule; li = labium; md = mandible; mx = maxilla.

**Figure 22:**
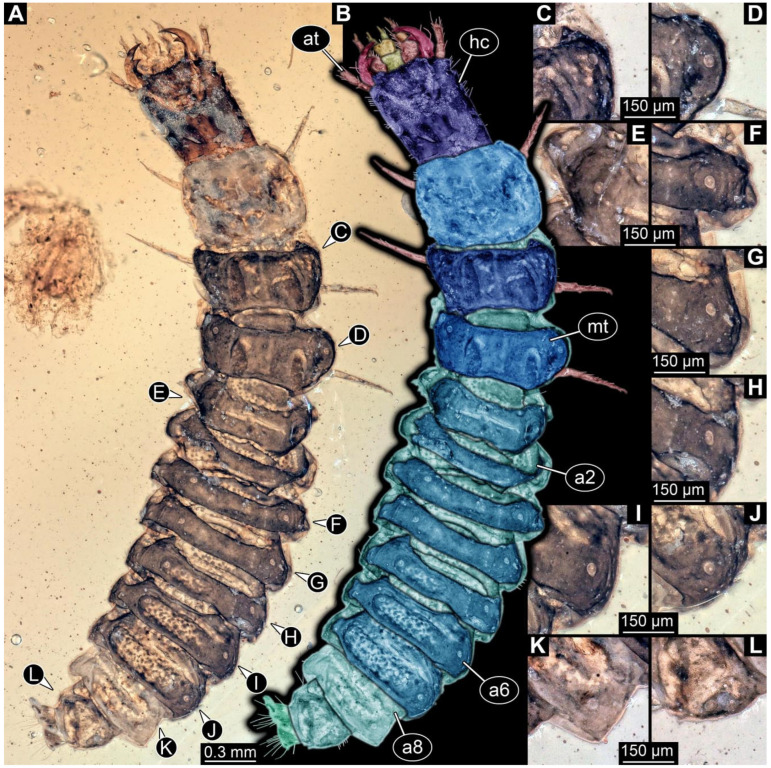
New soldier beetle larva from Kachin amber, Myanmar, BUB 3738. (**A**) Dorsal view. (**B**) Colour-marked version of (**A**). (**C**–**L**) Defensive glands details. Abbreviations: a2–8 = abdomen segment 2–8; at = antenna; hc = head capsule; mt = metathorax.

**Figure 23:**
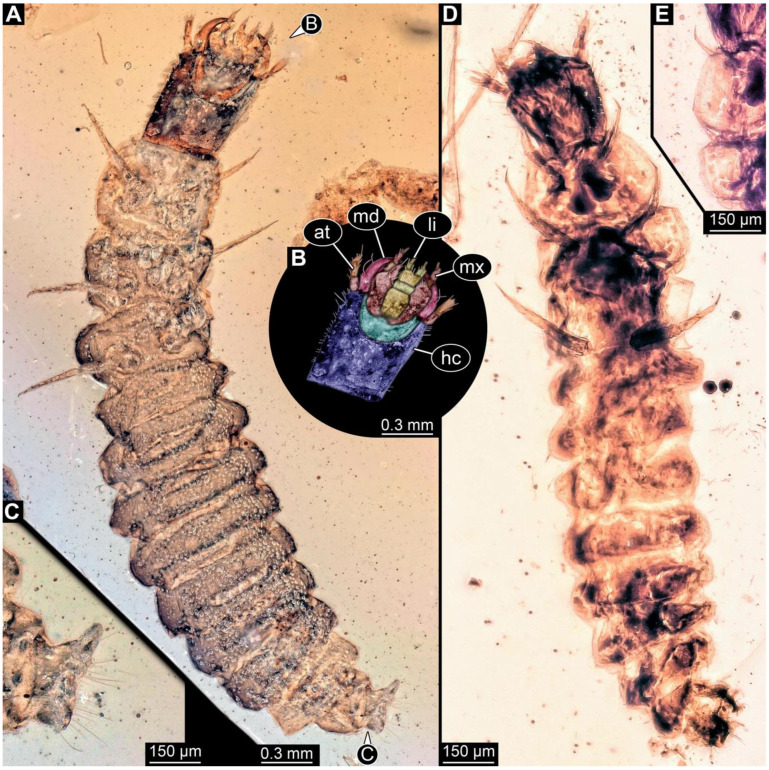
New soldier beetle larvae from Kachin amber, Myanmar. (**A**–**C**) BUB 3738, continued. (**A**) Ventral view. (**B**) Head detail, ventral view. (**C**) Trunk end (posterior end of abdomen). (**D**,**E**) BUB 5002. (**D**) Ventral view. (**E**) Defensive glands. Abbreviations: at = antenna; hc = head capsule; li = labium; md = mandible; mx = maxilla.

**Figure 24:**
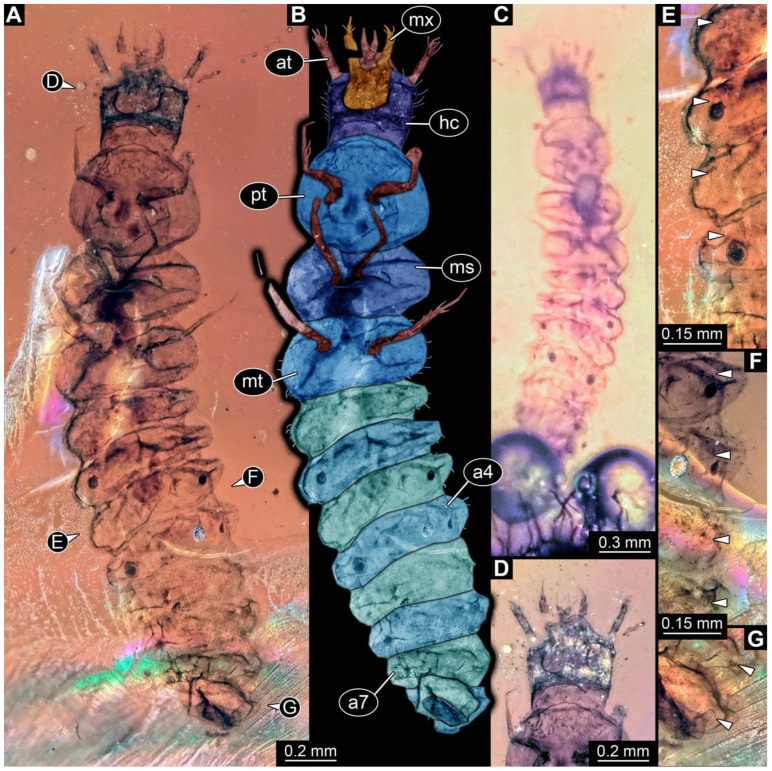
New soldier beetle larva from Kachin amber, Myanmar, PED 4125. (**A**) Ventral view. (**B**) Colour-marked version of (**A**). (**C**) Dorsal view. (**D**) Head detail, ventral view. (**E**–**G**) Defensive glands details, arrows mark defensive glands. Abbreviations: a4–7 = abdomen segment 4–7; at = antenna; hc = head capsule; ms = mesothorax; mt = metathorax; mx = maxilla; pt = prothorax.

**Figure 25:**
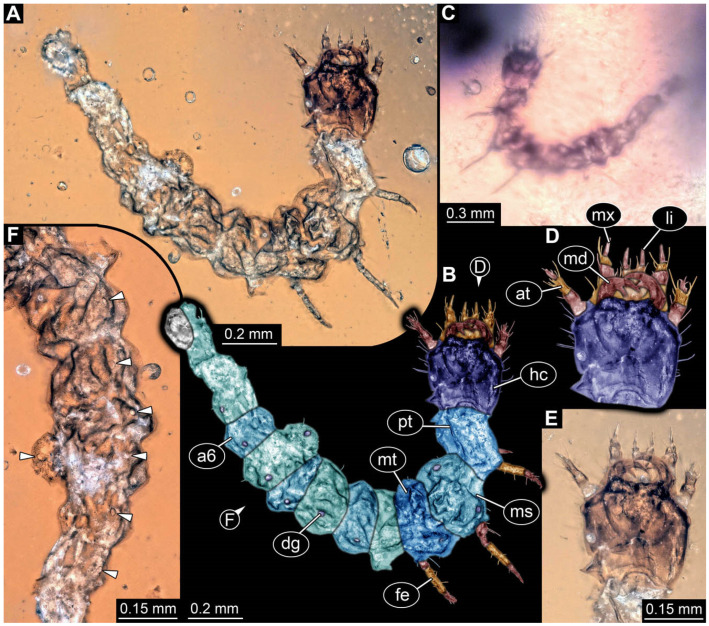
New soldier beetle larva from Kachin amber, Myanmar, PED 4179. (**A**) Dorsal view. (**B**) Colour-marked version of (**A**). (**C**) Ventral view. (**D**) Head detail, dorsal view, colour-marked version of (**E**). (**E**) Head detail, dorsal view. (**F**) Defensive glands detail, arrows mark defensive glands. Abbreviations: a6 = abdomen segment 6; at = antenna; dg = defensive gland; fe = femur; hc = head capsule; li = labium; md = mandible; ms = mesothorax; mt = metathorax; mx = maxilla; pt = prothorax.

**Figure 26:**
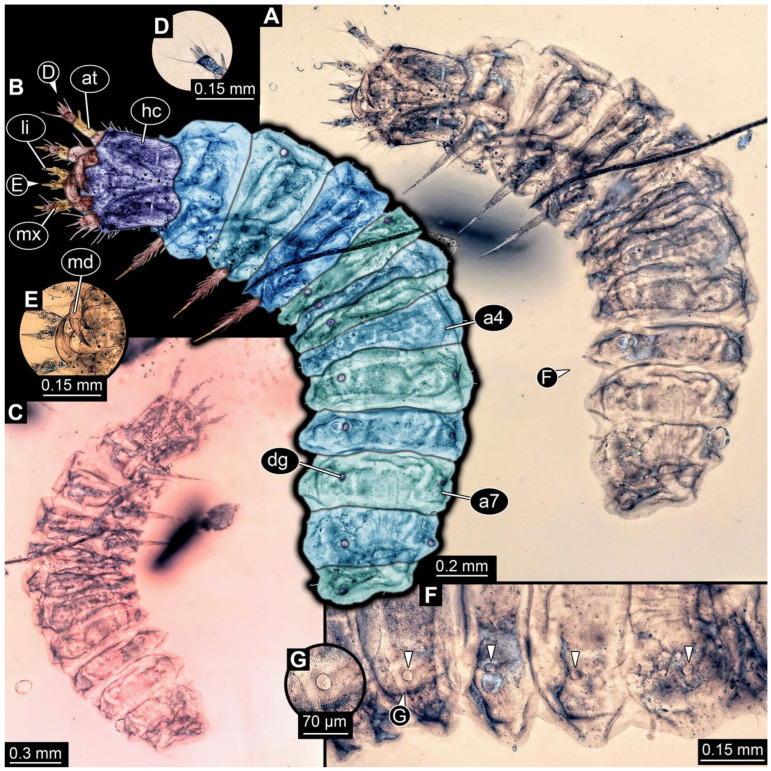
New soldier beetle larva from Kachin amber, Myanmar, PED 4274. (**A**) Dorsal view. (**B**) Colour-marked version of (**A**). (**C**) Ventral view. (**D**) Antenna detail. (**E**) Mandible detail. (**F**,**G**) Defensive glands details, arrows mark defensive glands. Abbreviations: a4 = abdomen segment 4; a7 = abdomen segment 7; at = antenna; dg = defensive gland; hc = head capsule; li = labium; md = mandible; mx = maxilla.

**Figure 27:**
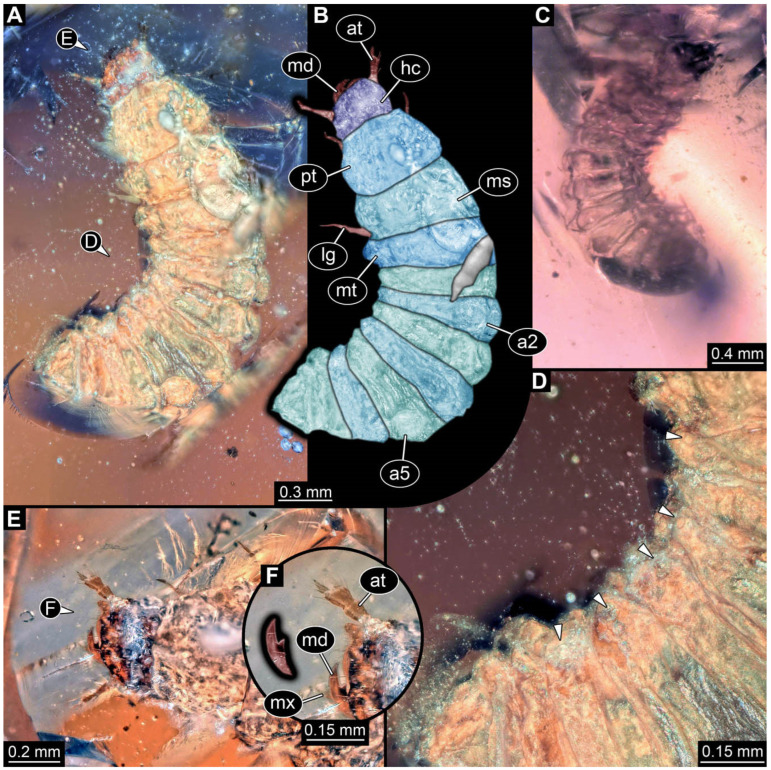
New soldier beetle larva from Kachin amber, Myanmar, PED 4291. (**A**) Dorsal view. (**B**) Colour-marked version of (**A**). (**C**) Ventral view. (**D**) Defensive glands detail, arrows mark defensive glands. (**E**) Head and prothorax detail. (**F**) Mouthparts details, colour-marked version of mandible. Abbreviations: a2–5 = abdomen segment 2–5; at = antenna; hc = head capsule; lg = leg; md = mandible; ms = mesothorax; mt = metathorax; mx = maxilla; pt = prothorax.

**Figure 28:**
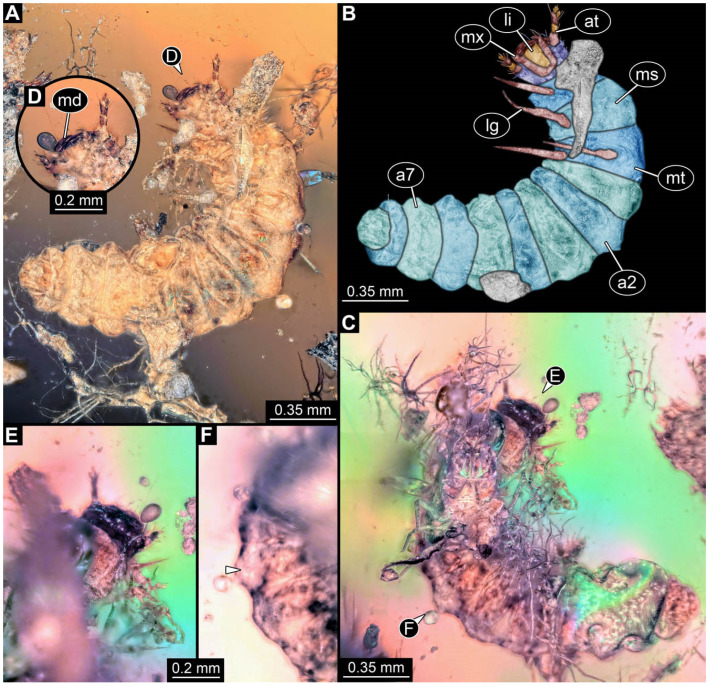
New soldier beetle larva from Kachin amber, Myanmar, PED 4311. (**A**) Ventral view. (**B**) Colour-marked version of (**A**). (**C**) Dorsal view. (**D**) Head detail, ventral view. (**E**) Head detail, dorsal view. (**F**) Defensive gland detail, arrow marks the defensive gland. Abbreviations: a2–7 = abdomen segment 2–7; at = antenna; lg = leg; li = labium; md = mandible; ms = mesothorax; mt = metathorax; mx = maxilla.

**Figure 29:**
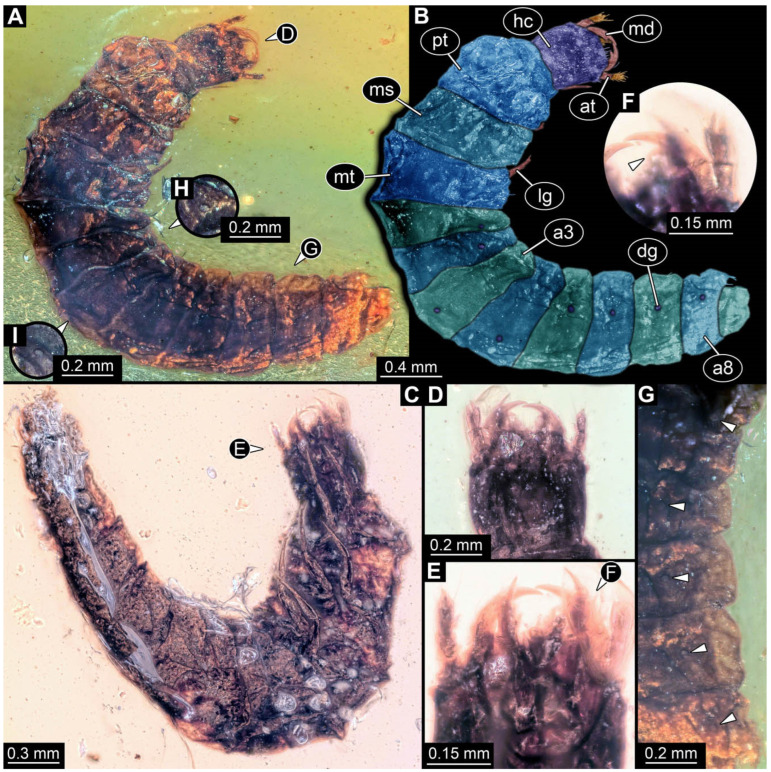
New soldier beetle larva from Kachin amber, Myanmar, PED 4312. (**A**) Dorsal view. (**B**) Colour-marked version of (**A**). (**C**) Ventral view. (**D**) Head detail, dorsal view. (**E**) Head detail, ventral view. (**F**) Mandible and antenna detail, arrow marks tooth. (**G–I**) Defensive glands details, arrows mark defensive glands. Abbreviations: a3–8 = abdomen segment 3–8; at = antenna; dg = defensive gland; hc = head capsule; lg = leg; md = mandible; ms = mesothorax; mt = metathorax; pt = prothorax.

**Figure 30:**
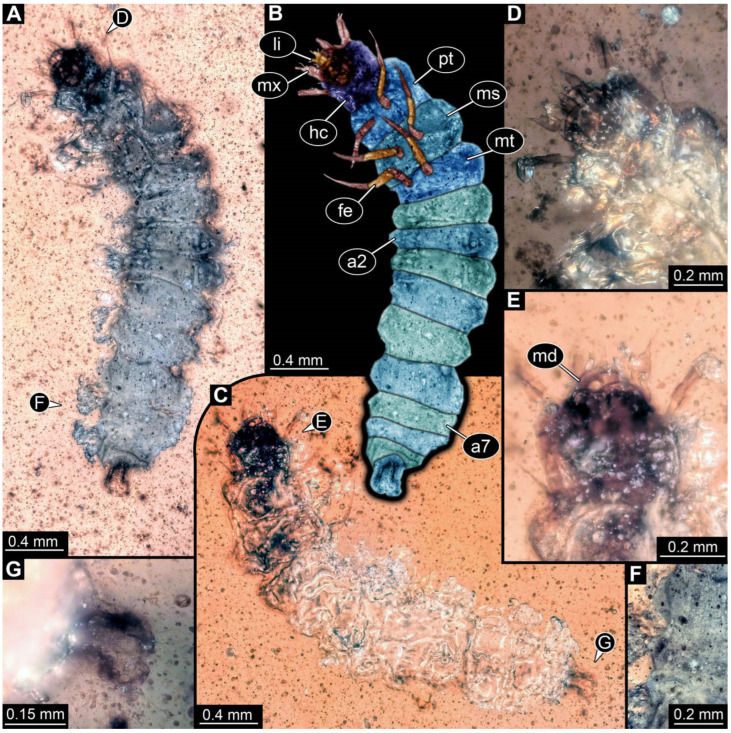
New soldier beetle larva from Kachin amber, Myanmar, PED 4358a. (**A**) Ventral view. (**B**) Colour-marked version of (**A**). (**C**) Dorsal view. (**D**) Head detail, ventral view. (**E**) Head detail, dorsal view. (**F**) Velvety surface detail. (**G**) Detail of trunk end (posterior end of abdomen). Abbreviations: a2–7 = abdomen segment 2–7; fe = femur; hc = head capsule; li = labium; md = mandible; ms = mesothorax; mt = metathorax; mx = maxilla; pt = prothorax.

**Figure 31:**
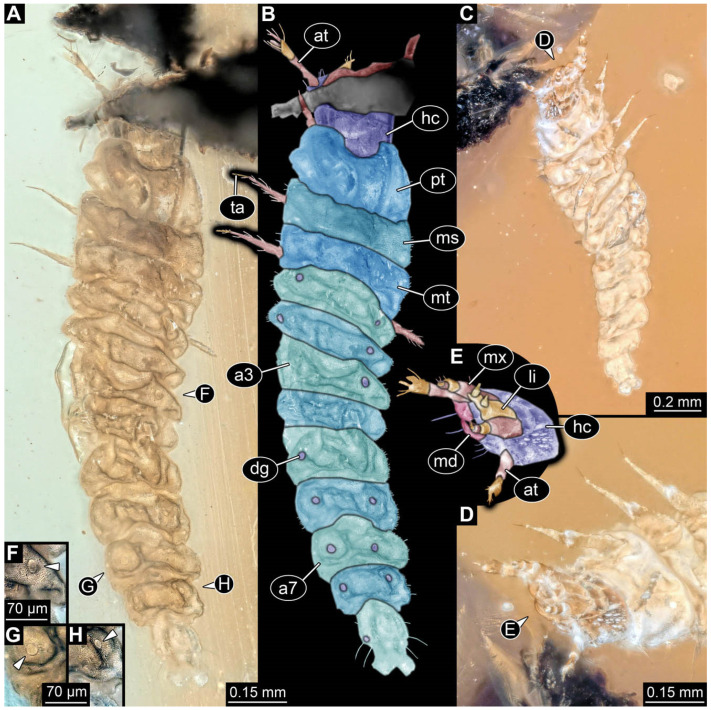
New soldier beetle larva from Kachin amber, Myanmar, PED 4394. (**A**) Dorsal view. (**B**) Colour-marked version of (**A**). (**C**) Ventral view. (**D**) Head detail, ventral view. (**E**) Colour-marked version of (**D**). (**F**–**H**) Defensive glands details, arrows mark defensive glands. Abbreviations: a3–7 = abdomen segment 3–7; at = antenna; dg = defensive gland; hc = head capsule; li = labium; md = mandible; ms = mesothorax; mt = metathorax; mx = maxilla; pt = prothorax; ta = tarsus.

**Figure 32:**
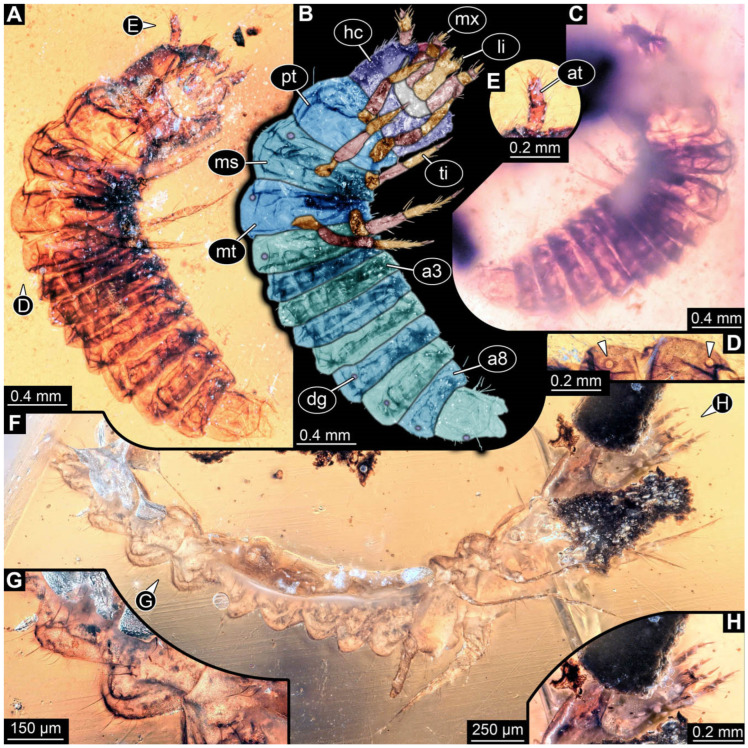
New soldier beetle larvae from Kachin amber, Myanmar. (**A**–**E**) BUB 4780b. (**A**) Ventral view. (**B**) Colour-marked version of (**A**). (**C**) Dorsal view. (**D**) Defensive glands detail, arrows mark defensive glands. (**E**) Antenna detail. (**F**–**H**) PED 2435. (**F**) Lateral view. (**G**) Velvety surface detail. (**H**) Head detail, ventro-lateral view. Abbreviations: a3–8 = abdomen segment 3–8; at = antenna; dg = defensive gland; hc = head capsule; li = labium; ms = mesothorax; mt = metathorax; mx = maxilla; pt = prothorax; ti = tibia.

**Figure 33:**
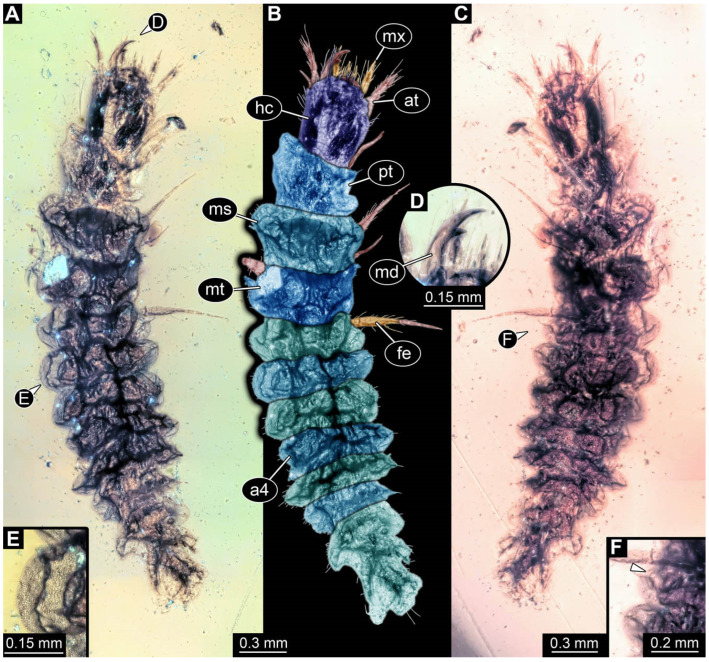
New soldier beetle larva from Kachin amber, Myanmar, BUB 5163. (**A**) Dorsal view. (**B**) Colour-marked version of (**A**). (**C**) Ventral view. (**D**) Mandible detail. (**E**) Velvety surface detail. (**F**) Defensive gland detail, arrow marks the defensive gland. Abbreviations: a4 = abdomen segment 3; at = antenna; fe = femur; hc = head capsule; md = mandible; ms = mesothorax; mt = metathorax; mx = maxilla; pt = prothorax.

The newly described specimens can be identified as beetles. Their mouthparts include a specific maxillo-labial complex, indicating the larvae are representatives of Elateroidea [4]. Within Elateroidea, they mostly resemble representatives of Cantharidae, especially in possessing openings indicating defensive glands and the highly abundant small setae over the surface, making the surface appear velvety. Most of the newly reported specimens show both of these features (Figure 2A–C,G,H, Figure 3C–J, Figure 4D–G, Figure 5B–K, Figure 6B, Figure 7A, Figure 10E, Figure 12E, Figure 13D,E, Figure 14C–G, Figure 15D–I, Figure 17D–G, Figure 18A,B, Figure 19C–F, Figure 20G–K, Figure 21D–L, Figure 22C–L, Figure 23E, Figure 24E–G, Figure 25F, Figure 26F,G, Figure 27D, Figure 28A,F, Figure 29G–I, Figure 31F–H, Figure 32D,G and Figure 33E,F) or at least one of these characters (Figure 2D,E, Figure 9A,C,E, Figure 10A,C,F,G, Figure 16C and Figure 30F). Only at specimens SNSB-BSPG 2018 III 61 (Figure 2I), Jonas Damzen, amberinclusions.eu, 13067 (Figure 6A), PED 0482_5 (Figure 8B), PED 0482_8 (Figure 8D), PED 2049 (Figure 11), and PED 3762 (Figure 16A,B), none of the two characteristics can be recognised due to preservation. Yet, all of the specimens fit the overall appearance of Cantharidae regarding, for example, body shape, and none of the specimens was an outlier in the analyses. Some specimens are only accessible ventrally (Figure 6A); hence, the possible defensive gland openings are not accessible. The overall habitus, as well as the accessible parts of the maxillo-labial complex, fit the morphology of larvae of Cantharidae. Two specimens are incomplete (Figure 8B,D); however, both specimens are preserved together with eight additional specimens of similar morphology in one amber piece that clearly show characters of Cantharidae. It seems likely that these were conspecific. PED 2049 (Figure 11A) is poorly preserved due to multiple bubbles and dirt on the surface of the specimen. The head resembles that of a soldier beetle larva. Further on, the anterior part of the prothorax, a small area not covered by dirt, can be recognised. There, a velvety surface can be seen, further supporting the interpretation as a larva of Cantharidae. The body of PED 3762 is partly disintegrated (Figure 16A). Hence, the defensive gland openings are not preserved. Some fractures of the surface can give the impression of a velvety surface. Also, the details of the head, especially the mandibles, resemble those of Cantharidae. Overall, we consider all larvae reported here to be soldier beetles.

### 4.2. Further Identification of the Larvae

Larvae of most extant ingroups of Cantharidae have maxilla palps with four elements (palpomeres; Cantharinae, Malthininae, Dysmorphocerinae, Silinae [5]). Only larvae of Chauliognathinae have just three elements (palpomeres). Of the newly described fossils with an accessible maxilla, only a few (six) specimens have maxilla palps with four elements (palpomeres; Figure 3B, Figure 7C,D, Figure 25D,E, Figure 26A,B, Figure 29E, and Figure 32H). All others, where accessible, clearly possess palps on the maxilla with three elements (palpomeres).

Also, only a few specimens (Figure 3B, Figure 6B, Figure 7B and Figure 29D) have a median projection on the anterior rim of the head capsule. This structure is absent in Chauliognathinae, but present in Cantharinae, Malthininae, Dysmorphocerinae, and Silinae [5]. Specimens with both structures available (maxilla palp and median projection) all have the combination of palps with four elements (palpomeres) and the median projection. These specimens are therefore likely not representatives of Chauliognathinae, but of another lineage. Unfortunately, other characters (e.g., details of defensive glands or mandibles) that would be necessary for a clearer interpretation are not easily accessible in these specimens.

The two characters (maxilla palps with three elements and absence of median projection) appear combined in most of the new specimens, indicating that most of the newly described fossils are larvae of the group Chauliognathinae or closely related to it. The previously reported fossil larva from Haug and Haug [13] was also discussed as being closely related to Chauliognathinae. Yet, the presence of a well-developed galea (endite) on the maxilla was observed in the fossil [13]. The galea is usually small (“minute”, [5] (table 3, p. 349)) in modern-day larvae of Chauliognathinae. This indicates that the larva reported by Haug and Haug [13] could be interpreted as an early representative of Chauliognathinae or its sister group.

In most of the newly described specimens, the region of the galea is not accessible. For these, we can not further elaborate on this aspect but conclude that they are possibly representatives of Chauliognathinae or a lineage closely related to it. For two specimens (BUB 3738, BUB 5163), we could identify a prominent galea (Figure 23A,B and Figure 33C). Therefore, these seem closely related to the specimen reported by Haug and Haug [13] and may be conspecifics. Five specimens (PED 0482_1, PED 0482_3; PED2699; PED 4077, BUB 4780b) have, in addition to the other characters shared with modern larvae of Chauliognathinae, short galeae (Figure 9A,B,E,F, Figure 13A,B, Figure 20B,C and Figure 32A,B). These are therefore very likely representatives of Chauliognathinae.

One specimen (PED 0321, Figure 7) represents a new combination of characters not known for larvae of Cantharidae so far. The galea is quite short (“minute”) as in modern larvae of Chauliognathinae (compare also [46] figures 16, 37, 58, 59, 68, 87, 104, 121, 147, 169, and 188), but the maxilla palps consist of four elements (palpomeres) and the head capsule shows a median projection. The galea would indicate an interpretation of the specimen as a representative of Chauliognathinae. However, the other two characters (median projection and number of elements of maxilla palp) clearly oppose the interpretation of the specimen being a representative of Chauliognathinae.

These differences indicate that among the larvae of Cantharidae preserved in amber, we already have quite some taxonomic diversity. It should not be surprising that not all of these larvae are conspecific, given the fact of a rather large record of adults of different species in amber [10].

### 4.3. Identity of New Specimen—Adult

**Figure 34:**
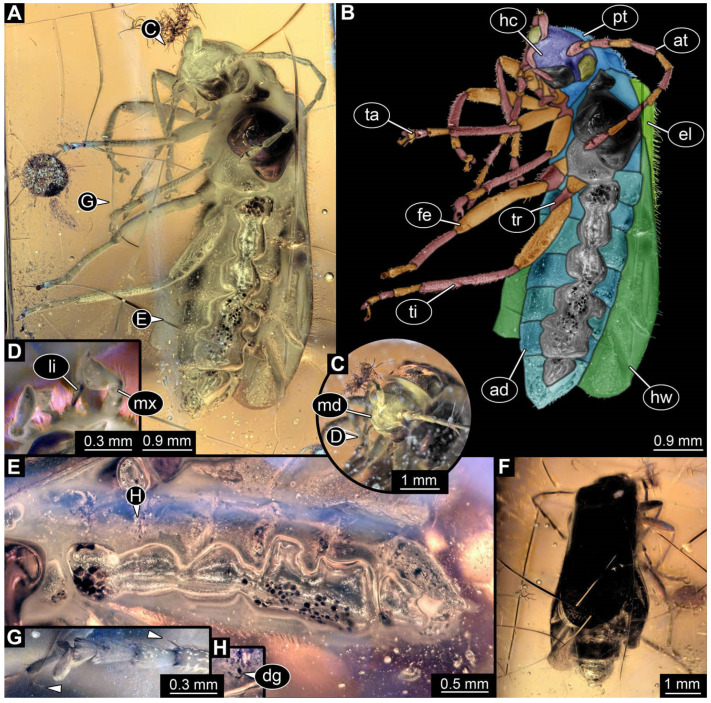
Adult soldier beetle from Baltic amber, SMNK-PAL 45612. (**A**) Ventral view. (**B**) Colour-marked version of (**A**). (**C**) Head detail, dorsal view. (**D**) Maxilla and labium detail. (**E**) Abdomen detail, lateral view. (**F**) Dorsal view. (**G**) Leg detail, arrows mark tibial spur and claw. (**H**) Defensive gland detail. Abbreviations: ad = abdomen; at = antenna; dg = defensive gland; el = elytra; fe = femur; hc = head capsule; hw = hindwing; li = labium; md = mandible; mx = maxilla; pt = prothorax; ta = tarsus; ti = tibia; tr = trochanter.

The body organisation and the presence of elytra clearly identify the specimen (SMNK-PAL 45612, Figure 34) as an adult of Coleoptera. Further, the specimen can be identified as a representative of Cantharidae based on various features shared with modern adult representatives. These features include the overall appearance (Figure 34A,B,F); a length of 7.86 mm, which is within the size range of modern representatives; a prognathous head with a membranous-appearing labrum (Figure 34C); the antenna with eleven elements (antennomeres; Figure 34A,B), as in modern adults of Cantharidae [8,18,82]; toothless, bent mandibles (Figure 34C; true for most modern adults); maxilla and labium of an overall shape similar to modern adults (although some details such as number of elements of palps are not accessible; Figure 34D); overall elongate and slender legs (Figure 34A,B); seemingly flattened coxae of the third leg pair (Figure 34A,B; [82]); and tarsi of all legs of the fossil specimen, each consisting of five elements (tarsomeres; Figure 34A,B), with the first three being rather similar (varying only in length), the fourth element being bilobed, and the last one bearing the claws (Figure 34G; [18,82]).

The exact number of abdomen segments is not clearly recognisable (Figure 34E). The specimen has at least seven abdomen units; six of these are abdomen segments, and the seventh is the undifferentiated trunk end. For modern adults, usually eight abdomen units have been recognised (seven abdomen segments + trunk end), but specimens with seven units (six abdomen segments + trunk end) have also been reported [82].

An additional trait always discussed for the determination of adults of Cantharidae is the soft body and weakly sclerotized elytra (which also earned them the German name “Weichkäfer” = “soft beetles”; [18]). However, this trait can not be clearly evaluated, since the specimen is preserved in amber.

Most importantly, lateral defensive glands are a diagnostic character to recognise adults of Cantharidae [82]. The fossil specimen also has openings resembling such defensive glands, even though not accessible on every segment (Figure 34E,H). Overall, this makes a strong case that this fossil is an adult soldier beetle.

Using the determination key of Ramsdale [82], the fossil specimen is likely a representative of Cantharinae and shows most similarities with representatives of the group *Rhagonycha.* Features supporting this interpretation include the head shape, shape of the distal palp element of the maxilla (Figure 34D), no modification of prothorax, a tibial spur (Figure 34G), the differentiation of the fourth tarsus element (Figure 34G), and elytra length (Figure 34F). The specimen is possibly a male, but the genitalia are not really visible. Further identification was not possible due to limited accessibility to other characters.

### 4.4. Fossil Record of Larvae of Cantharidae

Larvae of Cantharidae have already been reported in the fossil record (only three of them). One specimen was depicted by Janzen [11], but not determined as such, and one specimen each was reported by Fowler [12] and Haug and Haug [13]. Compared to the number of described adult specimens and species (around 200 fossil species [83]), the number of only three fossil larvae, previous to the current study, is remarkably small and indicates that soldier beetle larvae are rare fossils. The small number is also astonishing since soldier beetles (as well as many other beetles and, in fact, holometabolans) spend major parts of their lifetime as larvae (in the case of soldier beetles, 2–3 years [84]). Additionally, adults of Cantharidae have a quite short lifespan [18,85]. This leads to the expectation that larvae should be far more abundant than adult specimens. Also, the smaller size of the larvae should increase the probability of being entrapped and preserved in amber over the larger adult specimens [86,87,88,89]. The 45 new fossil soldier beetle larvae demonstrate that these fossils are not rare. This underpins that larvae of Cantharidae, as of other beetle groups, are not necessarily less abundant than adults, but rather shows that there is a reporting bias favouring adults.

Indeed, it would be important to also study the larvae, since this is a major phase of the lifetime of an insect, as well as the phase of growth; hence, the interaction in the ecosystem is far more intense during that phase. Therefore, larvae are crucial for a better understanding of ecosystems (see also discussion in Linhart et al. [29]). This study also supports the importance of larvae, as the results of the mandible analysis indicate a higher diversity in the diet of the larvae than in that of the adults (see also below). More data on larvae, fossil and extant, would be needed to increase our understanding of interactions in ecosystems.

### 4.5. Analyses—Pre-Larvae

**Figure 35:**
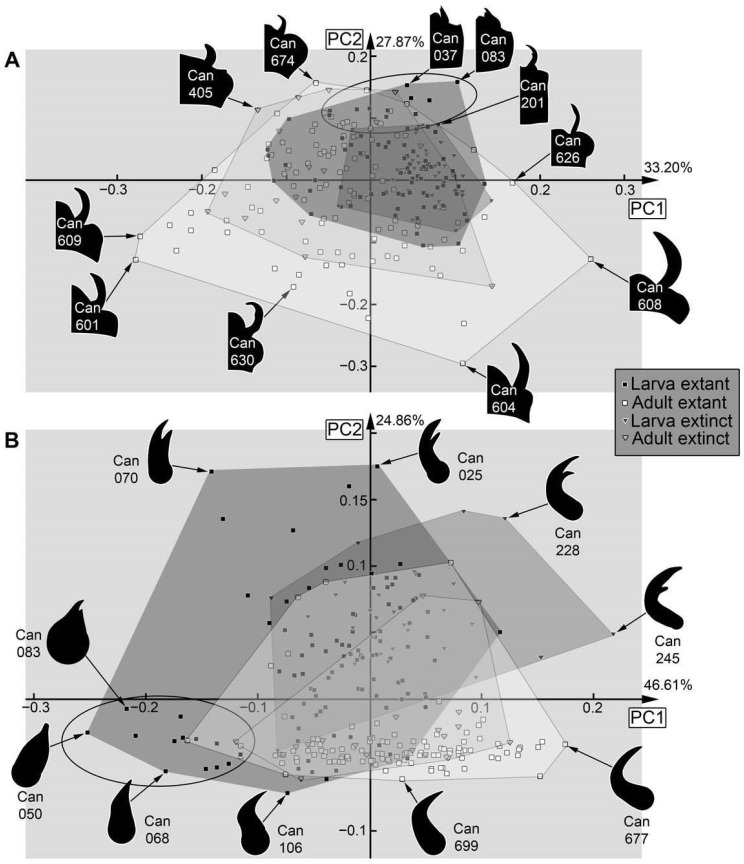
Morphospaces of shape analyses are represented by scatter plots of the principal components (PCs) 1 and 2. (**A**) Head capsule and mandible (01). (**B**) Mandible (02).

In several of the shape analyses, the pre-larvae can be recognised by their specific morphology. For example, in the head and mandible analysis (Figure 35A), all pre-larvae plot in the upper (right) corner. In the analysis of the mandibles, they are mostly separated, plotting in the lower left corner (Figure 35B), yet one supposed later larval specimen (Can_094, from [32]) plots together with the pre-larvae.

**Figure 36:**
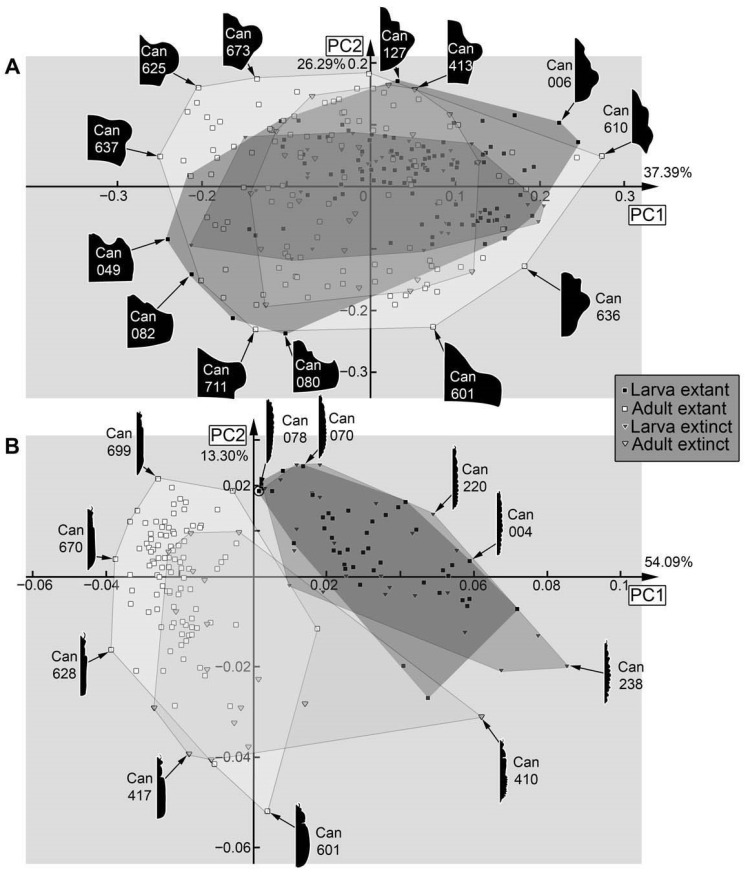
Morphospaces of shape analyses are represented by scatter plots of the principal components (PCs) 1 and 2. (**A**) Head capsule (03). (**B**) Full body with mandible (04).

**Figure 37:**
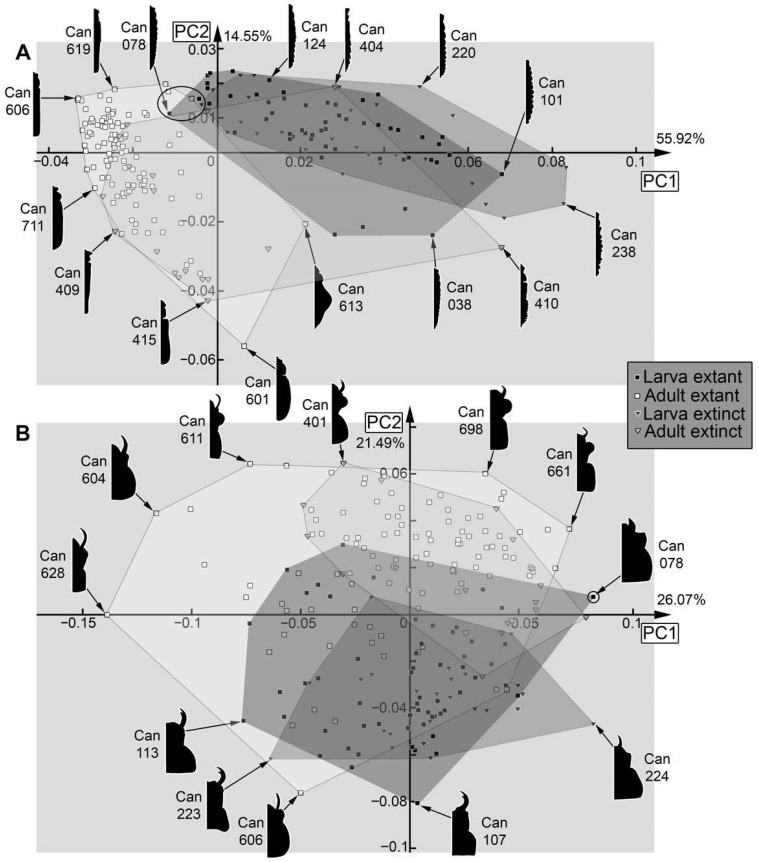
Morphospaces of shape analyses are represented by scatter plots of the principal components (PCs) 1 and 2. (**A**) Full body without mandible (05). (**B**) Head capsule, prothorax, and mandible (06).

**Figure 38:**
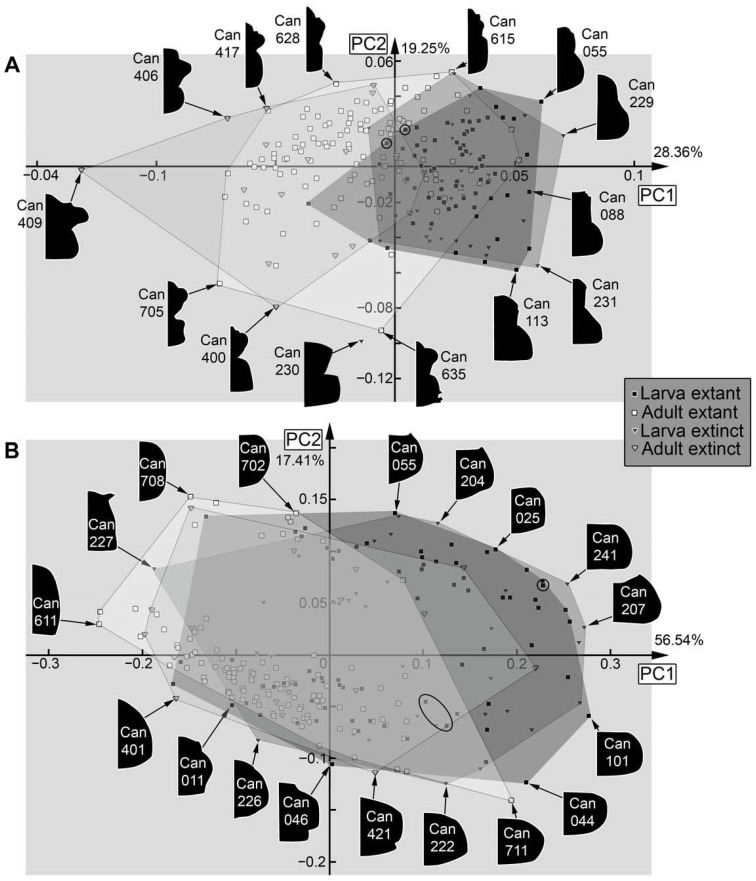
Morphospaces of shape analyses are represented by scatter plots of the principal components (PCs) 1 and 2. (**A**) Head capsule and prothorax (07). (**B**) Prothorax (08).

**Figure 39:**
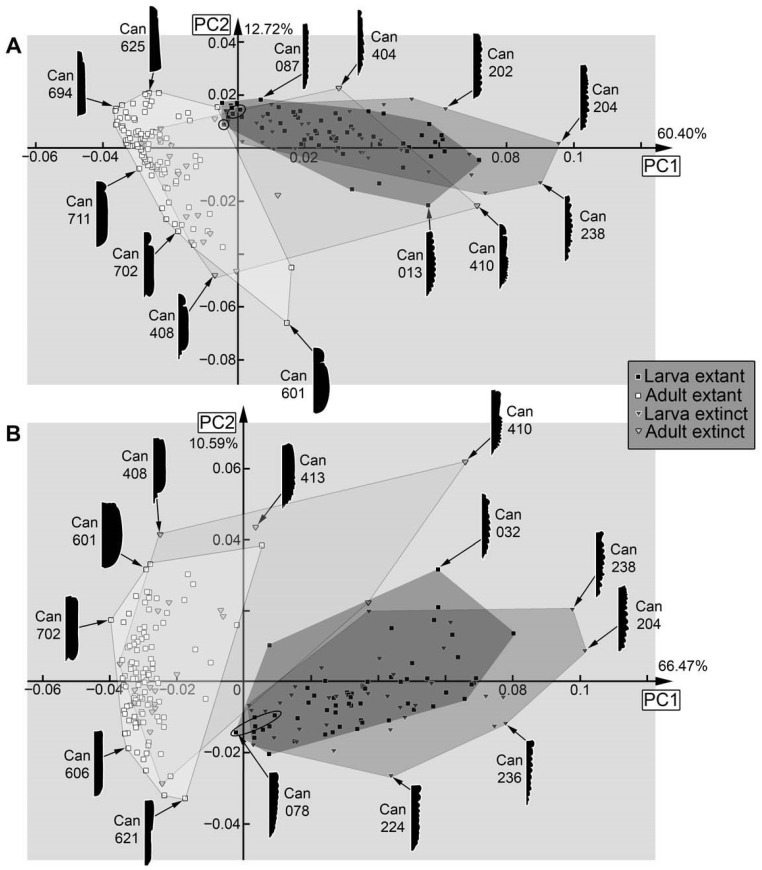
Morphospaces of shape analyses are represented by scatter plots of the principal components (PCs) 1 and 2. (**A**) Thorax and abdomen (=trunk) with prothorax (09). (**B**) Thorax and abdomen (=trunk) without prothorax (10).

**Figure 40:**
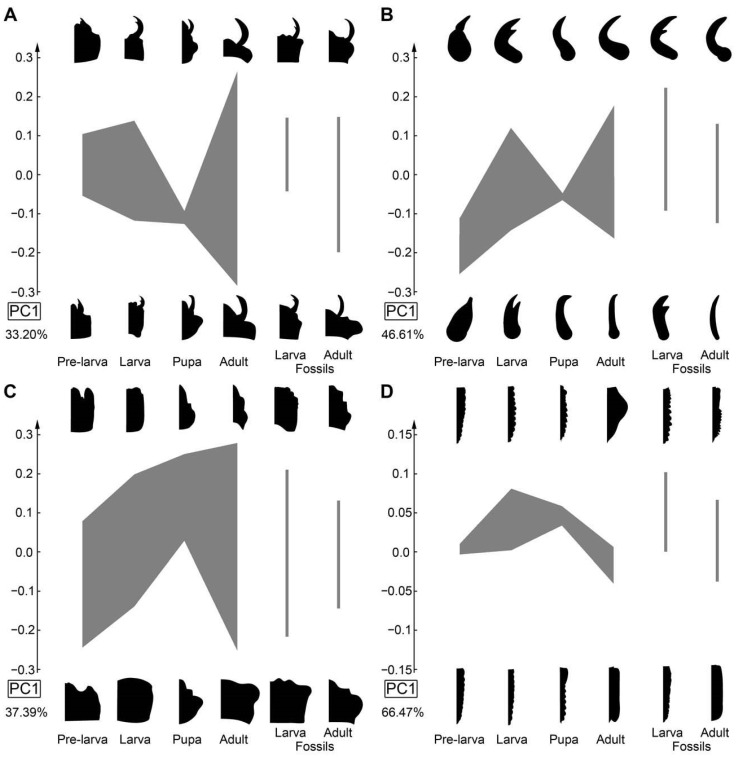
Morphospace of principal component 1 (PC1) vs. developmental stages considered (pre-larva, later larva, pupa, adult, fossil larva, and fossil adult). (**A**) Head capsule and mandible (01). (**B**) Mandible (02). (**C**) Head capsule (03). (**D**) Thorax and abdomen (=trunk) without prothorax (10).

Also in cases where the pre-larvae are not really separating from later larvae, they seem to expand the occupied area of the larvae, i.e., plot in extreme positions, as in the analyses of the head (Figure 36A), the entire body outline (Figure 36B), the body without mandibles (Figure 37A), head capsule, mandible, and prothorax (Figure 37B), and head capsule and prothorax (Figure 38A). In other aspects, they plot largely within the area occupied by the older larvae, as is the case for the prothorax (Figure 38B). The thorax and abdomen (=trunk) of pre-larvae are more similar to those of the adults, both in the analyses with prothorax (Figure 39A) and without the prothorax (Figure 39B). This is probably due to the yolk-filled and hence more swollen body of the pre-larvae being more similar to the even-shaped border of the elytra. However, also later larvae have bodies of a similar shape, and the pre-larvae plot more closely to (Figure 39B and Figure 40D) or are even covered completely by (Figure 39A) the later larvae and not together with the adults. These differences demonstrate that the head and mandible, and to a certain extent the thorax and abdomen (=trunk), are the structures that differentiate pre-larvae from later larvae. Overall, we have no indication for any fossil to represent a pre-larva, which is in line with the taxonomic aspects, as pre-larvae are only known in species of Cantharinae.

### 4.6. Analyses—Adults vs. Larvae

In several morphospaces, the area occupied by adults is larger than that of the larvae (Figure 37B and Figure 38A), even when pupae are grouped with larvae as they are immature (Figure 35A and Figure 36A). However, in none of the analyses was the area occupied by larvae completely covered by adults. Pupae plot further into the adults, as to be expected (Figure 35A, left side of larval morphospace).

In one case, the occupied area of the adults even almost embraces the occupied area of the larvae, namely for the head capsule (Figure 36A). The few specimens plotting outside the area of the adults are pre-larvae in the lower left and pupae in the upper right.

If looking at the mandible alone (Figure 35B), the larvae indeed occupy a larger area than the adults, indicating a larger variety in food sources. In this aspect, both groups also separate quite well. The main difference between larvae and adults is that the majority of the larvae have a tooth, while the majority of the adults do not. The pupae that could be included in this analysis did not expand the occupied area of the larvae. Also, the morphology of the prothorax (Figure 38B) seems to be more diverse in the larvae. The larvae distribute more equally over the morphospace, while adults mostly cluster together. This might reflect a stronger constraint on the adult prothorax.

In many other analyses, the larvae and adults strongly separate from each other, as is the case for the entire body including mandibles (Figure 36B), the entire body without the mandibles (Figure 37A), the thorax and abdomen (=trunk) with prothorax (Figure 39A), and without prothorax (Figure 39B). Also, in some of these separating cases, the adults occupy a larger area than the larvae do. Also, in the analysis of the head capsule and prothorax (Figure 38A), a slight separation can be observed. In this case, the area of the adults is clearly larger than that of the larvae.

### 4.7. Analyses—Fossil vs. Extant

Overall, fossils do (mostly) not plot outside the area occupied by the extant ones (e.g., Figure 37B); if they do, they do not deviate far, as is the case for head and mandible (Figure 35A), mandible (Figure 35B), or the prothorax (Figure 38B). One reason the extant larvae occupy a larger area than their fossil counterparts seems to be the pre-larvae, which are, so far, only known in the extant fauna (e.g., Figure 35A,B and Figure 37B); this can also be said for pupae (e.g., Figure 36A).

In some cases, the fossil and extant larvae occupy at most the more or less same area, as is the case for the full body (Figure 36B), the full body without the mandibles (Figure 37A), and the prothorax (Figure 38B). Yet, in the case of the full body without the mandibles (Figure 37A), a small shift is recognisable, with small areas only occupied by extant ones on one side and only fossil ones on the other side.

In the analysis of the full body without the mandibles (Figure 37A), fossil adults occupy a larger area than extant adults. This is mostly due to two fossil specimens (Can_404; Can_410); these have some characters of the larvae, the elytra are shorter, revealing the posterior abdomen. While there are also extant specimens with short elytra, it seems to affect the overall outline more strongly for the fossils. Also, other larva-like adults are recognisable in the dataset of thorax and abdomen (=trunk) (Figure 39A,B).

For the head and prothorax, a single fossil adult (Can_409) plots at an extreme position. This specimen has extremely exposed eyes, which are not present in any other specimen.

In general, we can not recognise major changes in morphological diversity over time. The only difference appears to be the presence of pre-larvae in the extant fauna, which are absent among the fossils.

### 4.8. Hypermetamorphosis

Hypermetamorphosis was already discussed in Cantharidae by Verhoeff [14]. He concluded that Cantharidae do not have a hypermetamorphic development because they do not undergo a “step backward” [14] (p. 138). Further, he described the pre-larvae as preparation for the later larvae and named the development “Fötomorphose” (≈“Embryomorphosis”; [14] (p. 138)). However, this description and interpretation were based only on qualitative aspects, and with the distinct pre-larvae, they indeed fulfil an important criterion of hypermetamorphosis: the larval phase includes two distinct sub-phases, in which the larvae of the earlier sub-phase differ morphologically and ecologically from the larvae of the later one.

Quantitatively, we have expected two aspects: (1) a clear separation of pre-larvae from later larvae, and (2) an “indirect pattern” with later larvae differing more strongly from adults than early ones [90].

No clear signal of hypermetamorphosis could be found. In most analyses, the pre-larvae show a certain difference to the later larvae but are mostly close to them, often plotting among the later larvae (e.g., Figure 40A). The most distinct separation of the pre-larvae from the other specimens can be found in the analysis of the mandible (Figure 35B and Figure 40B). Also, regarding the overall developmental trajectory, it does not appear “indirect”, as pre-larvae often plot at more extreme positions and further away from the adults than later larvae, more or less forming a direct type of developmental trajectory (Figure 40B,D). Although it may look in some cases like there could be a distinct kink in the trajectories (Figure 40), these seem mostly coupled to the lower variation seen in the pupa stage, which is very likely caused by the very low sample size for this sub-group. The overall observation fits the interpretation of Verhoeff [14], with the pre-larvae showing a morphology that is in a simple trajectory towards later larvae and adults. Considering the herein conducted quantitative analysis, the results do not indicate that soldier beetles undergo hypermetamorphosis.

An additional aspect that could be interpreted as an argument against hypermetamorphosis in Cantharidae is the shortening of the elytra in many species. Shorter elytra make the specimens appear more larva-like, as also apparent in the shape analysis (Figure 39B). Ecologically, this wing shortening allows the adults to enter areas that otherwise are only available to the larvae with their more movable abdomen (see also discussion in [91] on Staphylinidae). A smaller change from larva to adult would represent a less strong metamorphosis in outer morphology instead of a stronger one. However, this may not exclude a hypermetamorphic development. Quite some groups that are considered hypermetamorphic have paedomorphic (≈ larviform) females (e.g., Strepsiptera: [92]; Drilini: [93,94]; Ripiphoridae: [95,96]). This indicates that a smaller change from larva to adult does not exclude hypermetamorphosis.

### 4.9. No Losses in Beetles?

When looking at quantitative comparisons including animals from Kachin amber, we can recognise losses of morphological diversity over time in some neuropteriformian groups (e.g., Raphidioptera [97]; Neuroptera, e.g., [76,77,98,99], but very little so in beetles to date (minor examples in: [100] for Elateridae; [101] for Dermestidae). For many beetle groups, the morphological diversity of the larvae seems either to have remained rather similar ([102] for Texas beetles), or we can even recognise an increase in morphological diversity after the Cretaceous ([91] for rove beetles). Also, for soldier beetles, it looks like the modern fauna has more morphological variation than the fossil record. While there are slight shifts indicated here and there, we see nothing major standing out, indicating a very different morphology being lost. Instead, it appears that the morphology of the pre-larvae indeed represents a new one that evolved only after the Cretaceous. However, it can not be said for sure if the pre-larvae were truly absent or have not been reported yet. We could so far assume that there is a certain stability in the morphological diversity of beetle larvae, but this might be due to a simple lack of data. Analyses such as those performed here seem to indeed support such an assumption.

## Data Availability

All data from this study are available in this paper and the associated papers.

## Supplementary Materials

The following supporting information can be downloaded at https://www.mdpi.com/article/10.3390/insects17040406/s1. Supplementary Table S1: Information on the specimens investigated in this study and used for the shape analyses. Supplementary Table S2: Descriptive matrix for the new specimens depicted in this paper, X = present; − = absent; ? = unclear. Supplementary File S1: Results of the shape analyses in detail. Figure 1, Figure 2, Figure 3, Figure 4, Figure 5, Figure 6, Figure 7, Figure 8, Figure 9, Figure 10, Figure 11, Figure 12, Figure 13, Figure 14, Figure 15, Figure 16, Figure 17, Figure 18, Figure 19, Figure 20, Figure 21, Figure 22, Figure 23, Figure 24, Figure 25, Figure 26, Figure 27, Figure 28, Figure 29, Figure 30, Figure 31, Figure 32, Figure 33 and Figure 34 in high resolution are available at https://doi.org/10.5281/zenodo.18185001 (accessed on 1 March 2026).
