# Supplementary material for: Soldier Beetle Larvae Are Much More Common in the Fossil Record than Previously Anticipated
Source: Insects. 2026 Apr 9;17(4):406. doi: 10.3390/insects17040406 (PMC13116794; doi:10.3390/insects17040406)
Supplement: Supplementary file 1 [file insects-17-00406-s001.zip › Supplement_Linhart rev/Supplementary_FileS1.zip!/Supplementary_FileS1/05.pdf]

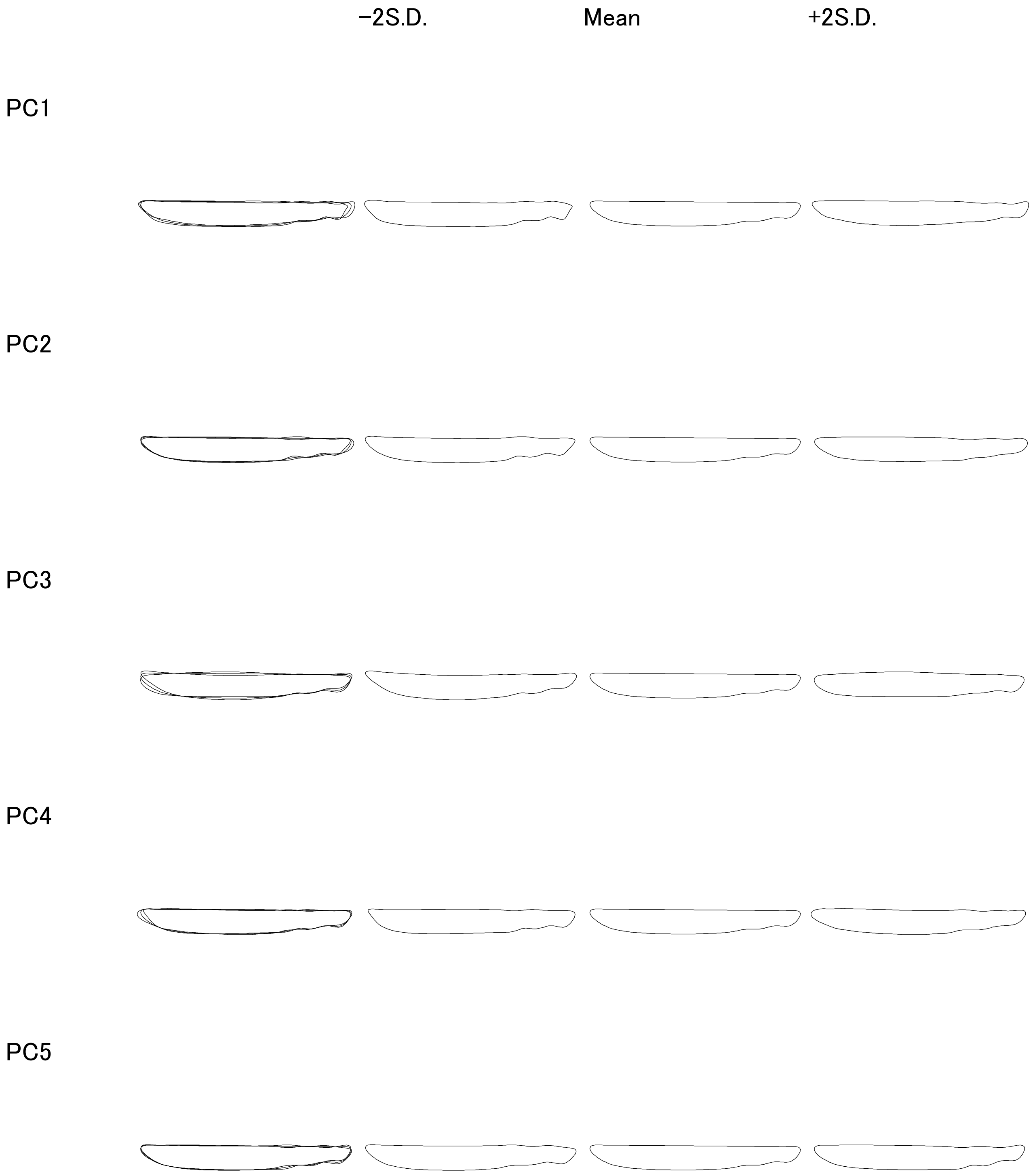
−2S.D.
Mean
+2S.D.
PC1
PC2
PC3
PC4
PC5

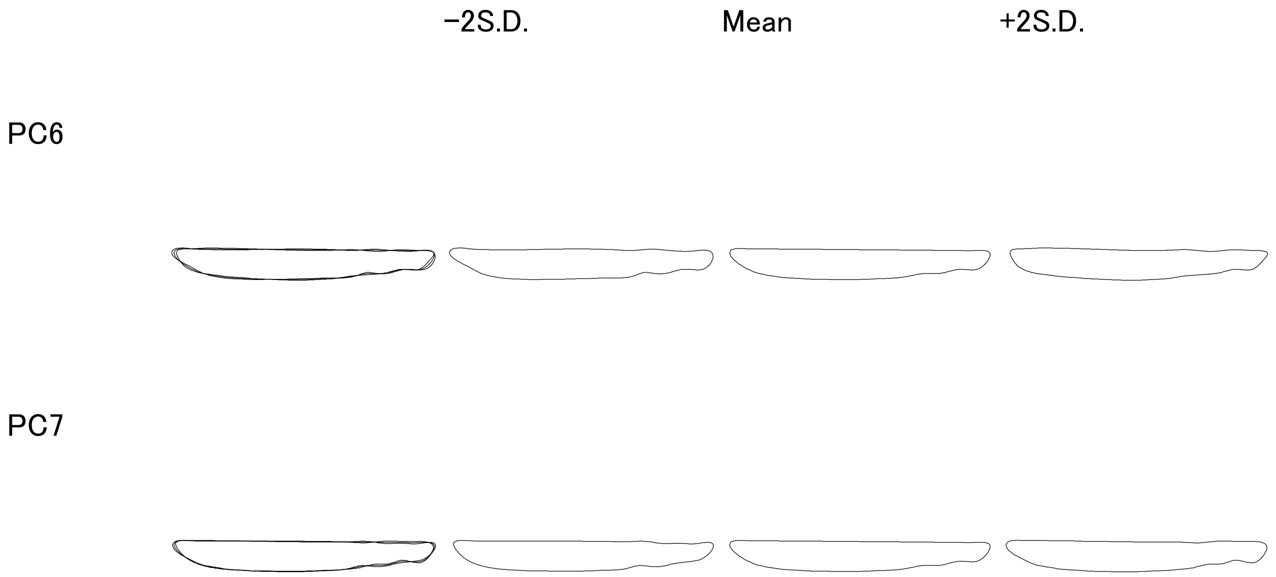
−2S.D.
Mean
+2S.D.
PC6
PC7
